# Heterogeneity of Astrocytes: From Development to Injury – Single Cell Gene Expression

**DOI:** 10.1371/journal.pone.0069734

**Published:** 2013-08-05

**Authors:** Vendula Rusnakova, Pavel Honsa, David Dzamba, Anders Ståhlberg, Mikael Kubista, Miroslava Anderova

**Affiliations:** 1 Laboratory of Gene Expression, Institute of Biotechnology, Academy of Sciences of the Czech Republic, Prague, Czech Republic; 2 Department of Cellular Neurophysiology, Institute of Experimental Medicine, Academy of Sciences of the Czech Republic, Prague, Czech Republic; 3 Sahlgrenska Cancer Center, Department of Pathology, Sahlgrenska Academy at the University of Gothenburg, Gothenburg, Sweden; University of Louisville, United States of America

## Abstract

Astrocytes perform control and regulatory functions in the central nervous system; heterogeneity among them is still a matter of debate due to limited knowledge of their gene expression profiles and functional diversity. To unravel astrocyte heterogeneity during postnatal development and after focal cerebral ischemia, we employed single-cell gene expression profiling in acutely isolated cortical GFAP/EGFP-positive cells. Using a microfluidic qPCR platform, we profiled 47 genes encoding glial markers and ion channels/transporters/receptors participating in maintaining K^+^ and glutamate homeostasis per cell. Self-organizing maps and principal component analyses revealed three subpopulations within 10–50 days of postnatal development (P10–P50). The first subpopulation, mainly immature glia from P10, was characterized by high transcriptional activity of all studied genes, including polydendrocytic markers. The second subpopulation (mostly from P20) was characterized by low gene transcript levels, while the third subpopulation encompassed mature astrocytes (mainly from P30, P50). Within 14 days after ischemia (D3, D7, D14), additional astrocytic subpopulations were identified: resting glia (mostly from P50 and D3), transcriptionally active early reactive glia (mainly from D7) and permanent reactive glia (solely from D14). Following focal cerebral ischemia, reactive astrocytes underwent pronounced changes in the expression of aquaporins, nonspecific cationic and potassium channels, glutamate receptors and reactive astrocyte markers.

## Introduction

Astrocytes comprise a heterogeneous cell type with several subgroups; for review see [1]. Even within the same brain region multiple astrocytic subgroups have been observed [2]. In addition to morphological differences, astrocytes show diversity in Ca^2+^ signalling, gap junction coupling, and the expression of membrane proteins such as K^+^ channels, glutamate receptors and transporters; for review see [3]. It was recently shown that astrocyte heterogeneity also rests on the expression of inwardly rectifying (KIR) and two-pore-domain K^+^ (K2P) channels [4]. In our recent work we demonstrated the presence of two distinct subpopulations of astrocytes that respond differently to oxygen-glucose deprivation, probably due to their different expression of chloride channels (ClC2), inwardly rectifying K^+^ channels (KIR4.1) and K2P channels, such as TREK-1 and TWIK-1 [5]. Astrocytes also change their properties during development. In contrast to neurons, astrocytes are formed at late stages of embryogenesis (from E16 and onward) and during the first postnatal weeks [6], and the mouse cortex is not fully developed until between the 3^rd^ and 4^th^ week after birth [7]. The origin of astrocytes is not fully known; possibly they arise from distinct groups of progenitors [8], and some subpopulations may be generated from NG2 glia cells [9]. NG2 glia are characterized by their expression of NG2 chondroitin sulphate proteoglycan (CSPG4) and platelet-derived growth factor α receptor (PDGFαR). After their discovery, NG2 glia were found to be oligodendrocyte progenitor cells [10]. Later, NG2 glia were also shown to be progenitors of some groups of reactive astrocytes [11], [12], which appear after CNS injury. This process, termed astrogliosis, is characterized by the increased expression of glial fibrillary acidic protein (GFAP) and Nestin, followed by the elevated expression of vimentin [13]. Another characteristic of astrogliosis is the high expression of aquaporins, especially of AQP1 and AQP9, which are proteins rarely expressed in healthy tissue [14], [15]. Several publications have reported reactive astrocytes expressing proteoglycans [16], [17] and glutamate metabotropic receptor 5 (*Grm5*) [18], [19]. Traditionally, astrocytes are identified by the expression of GFAP [20]. However GFAP is not always expressed in non-reactive astrocytes, and GFAP is frequently not immunohistochemically detected in astrocytes in healthy tissue [21]. Other complementary markers are therefore used to identify astrocytes, including S100β [22], excitatory amino acid transporter 1 and 2 (EAAT1 and 2) [23], glutamine synthetase (GS) [24] and aldehyde dehydrogenase 1 family member L1 (ALDH1L1) [25]. Since non-glial cells also express some of these markers, detecting a combination of markers is needed for the reliable identification of astrocytes [26].

Altogether, there is evidence of astrocyte heterogeneity between, as well as within, CNS regions. To characterize the different types of astrocytes that appear under various conditions, single-cell studies are necessary. Single cell gene expression profiling is a novel and powerful method to study cell heterogeneity [27]. It has already been used to establish the presence of distinct subpopulations of astrocytes in a seemingly homogenous macroscopic population [5], [28]. Single cell expression profiling is challenging because of the limited amount of RNA present in a single cell. Very efficient reverse transcription is needed, and for the analysis of more than some 10 genes pre-amplification is required. Cells express mRNAs in bursts [29], [30], resulting in large natural variation in the number of any transcript among cells, which can be modelled with a lognormal distribution [31]. To obtain biologically relevant and statistically significant conclusions, rather large numbers of cells must be analysed [32]. A particularly powerful approach to distinguish different cells and subtypes of cells is to study the correlation of transcript levels within individual cells [33]. This kind of information is not available when studying classical samples composed of many cells.

Our goal was to characterize astrocytes based on changes in their gene expression profiles throughout development and after focal cerebral ischemia. We used single cell RT-qPCR profiling to analyse EGFP-positive (EGFP^+^) cells collected by fluorescence-activated cell sorting (FACS) from the cortex of GFAP/EGFP transgenic mice with the objective of identifying and characterizing subpopulations of astrocytes. We focus our profiling effort on glial glutamate transporters and receptors as well as on ion channels, which are critical for the maintenance of the ionic/neurotransmitter homeostasis of neurons and glial cells under physiological conditions as well as in pathological states.

## Materials and Methods

### Induction of an Ischemic Lesion in Adult GFAP/EGFP Mice

All experiments were performed on cells from acutely isolated brains of GFAP/EGFP transgenic mice [line designation TgN(GFAPEGFP)], in which the expression of EGFP is controlled by the human GFAP promoter [34]. All procedures involving the use of laboratory animals were performed in accordance with the European Communities Council Directive 24 November 1986 (86/609/EEC) and animal care guidelines approved by the Institute of Experimental Medicine, Academy of Sciences of the Czech Republic (Animal Care Committee on April 17, 2009; approval number 85/2009).

Prior to the induction of focal ischemia, 50-day-old (P50) mice were anaesthetized with 1.5% isoflurane (Abbot) and maintained in 1% isoflurane using a vaporizer (Tec-3, Cyprane Ltd., Keighley). A skin incision between the orbit and the external auditory meatus was made, and a 1–2 mm hole was drilled through the frontal bone 1 mm rostral to the fusion of the zygoma and the squamosal bone and about 3.5 mm ventral to the dorsal surface of the brain. The middle cerebral artery (MCA) was exposed after the dura was opened and removed. The MCA was occluded by short coagulation with bipolar tweezers (SMT, Czech Republic) at a proximal location, followed by transection of the vessel to ensure permanent occlusion. The mice received 0.5 ml saline solution subcutaneously, and during the surgery the body temperature was maintained at 37±1°C using a heating pad.

### Immunohistochemistry

The mice were deeply anaesthetized with pentobarbital (PTB, 100 mg/kg, i.p.) and perfused transcardially with 20 ml of saline followed by 20 ml of cooled 4% paraformaldehyde in 0.1 M phosphate buffer. Brains were dissected out, fixed with paraformaldehyde overnight, cryoprotected with sucrose and sliced into 30 µm coronal slices using a microtome (HM550, Microm International). The slices were incubated with 5% Chemiblocker (Millipore) and 0.2% Triton in PBS then with the primary antibodies at 4°C overnight. As primary antibodies, we used anti-GFAP (1∶800, Sigma-Aldrich, mouse, conjugated with Cy3), anti-GFAP (1∶800, Sigma-Aldrich, mouse), anti-NG2 (1∶400, Chemicon, rabbit) and anti-PDGFαR (1∶200, Santa Cruz, rabbit). Finally, the secondary antibody was applied for 2 hours, either goat anti-mouse IgG or goat anti-rabbit IgG conjugated with Alexa Fluor 660 (Molecular Probes). A Zeiss 510DUO LSM confocal microscope equipped with Arg/HeNe lasers was used for immunohistochemical analysis. Colocalization images and their maximum *z* projections were made using a Zeiss LSM Image Browser.

### Preparation of Cell Suspensions from the Cortex of GFAP/EGFP Mice

GFAP/EGFP transgenic mice 10, 20, 30 and 50 days old (P10, P20, P30, P50) or 3, 7, and 14 days after middle cerebral artery occlusion (MCAO) (D3, D7, D14) were deeply anesthetized with PTB (100 mg/kg, i.p.) and perfused transcardially with cold (4–8°C) isolation buffer containing (in mM): NaCl 136.0, KCl 5.4, Hepes 10.0, glucose 5.5, osmolarity 290±3 mOsmol/kg. The forebrain was isolated by the removal of the olfactory lobes, cerebellum, and midbrain/hindbrain structures by dissection. To isolate the cerebral cortex, the brain (+2 mm to −2 mm from bregma) was sliced into 400 µm coronal sections using a vibrating microtome HM650V (MICROM International GmbH), and the dorsal cerebral cortex was carefully dissected out from the ventral white matter tracks. The tissue was incubated with continuous shaking at 37°C for 90 minutes in 5 ml of a papain solution (20 U/ml) and 0.2 ml DNase (both from Worthington) prepared in isolation buffer. After papain treatment, the tissue was mechanically dissociated by gentle trituration using a 1 ml pipette. Dissociated cells were layered on the top of 5 ml of Ovomucoid inhibitor solution (Worthington) and harvested by centrifugation (140×g for 6 minutes). This method routinely yielded ∼2×10^6^ cells per mouse. Cell aggregates were removed by filtering with a 30 µm cell strainer (Becton Dickinson), and the cells were kept on ice until sorting.

### Collection of Single EGFP^+^ Cells

Single cells were sorted using flow cytometry (BD Influx). The flow cytometer was manually calibrated to deposit a single cell in the centre of each collection tube. Hoechst 33258 (Life Technologies) was added to the suspension of cells to check viability. Initially, cell sorting was performed with a negative control (no fluorescent cells) in order to set the fluorescence threshold for collecting EGFP^+^ cells and to avoid auto-fluorescent cells. After setting the threshold, all EGFP^+^ cells that crossed this level of fluorescence were collected. Single cells were collected into 96-well plates (Life Technologies) containing 5 µl nuclease-free water with bovine serum albumin (1 mg/µl, Fermentas) and RNaseOut 20 U (Life Technologies). Plates were placed on a pre-cooled rack. The glial cells collected were positive for EGFP and viable. Collected cells in 96-well plates were immediately placed at −80°C. For analysing individual developmental or post-ischemic stages, the cells were isolated from 1 mouse (P10), 2 mice (P20), 1 mouse (P30), 1 mouse (P50), 2 mice (D3), 2 mice (D7) and 1 mouse (D14).

### cDNA Synthesis

cDNA synthesis was performed with SuperScript III RT (Life Technologies). Lysed single cells in 5 µl water containing 0.5 µM dNTP (Promega), 1.0 µM oligo (dT15) (Invitrogen) and 1.0 µM random hexamers (Eastport) were incubated at 70°C for 5 min. 50 mM Tris–HCl, 75 mM KCl, 3 mM MgCl_2_, 5 mM dithiothreitol, 20 U RNaseOut and 100 U SuperScript III (all Life Technologies) were added to a final volume of 10 µl. Reverse transcription was performed at 25°C for 5 min, then at 50°C for 60 min, followed by 55°C for 15 min and finally terminated by heating at 70°C for 15 min. Four µl from each sample was diluted 4 times and used for pre-testing. Five µl of cDNA was used for preamplification.

### qPCR

Primers were designed using BeaconDesigner (version 7.91, Premier Biosoft International) as previously described [5]. Primer sequences are provided in Table S1. All single cells were pre-tested for the expression of glutamate transporter (*Eaat2*), glutamine synthetase (*Glul*), which are markers of astrocytes, and chondroitin sulphate proteoglycan (*Cspg4*) and platelet-derived growth factor α receptor (*Pdgfra*), which are markers of NG2 glia, to select samples for further gene expression profiling using assays for 47 genes. A CFX384 (Biorad) was used for all qPCR measurements. To each reaction (10 µl) containing iQ SYBR Green Supermix (BioRad) and 400 nM of each primer (EastPort), we added 3 µl of diluted cDNA. The temperature profile was 95°C for 3 min followed by 50 cycles of amplification (95°C for 15 s, 60°C for 15 s and 72°C for 20 s). All samples were subjected to melting curve analysis. The same experimental setup was used to test preamplification.

### Preamplification and qPCR

The applied preamplification protocol was verified on samples from three animals. The RNA was extracted and cDNA was prepared to test the preamplification protocol. Each reaction contained 25 µl of iQ Supermix (BioRad), 5 µl of a mix of all primers (final concentration 25 nM each), 5 µl of non-diluted cDNA, and water added to a final volume of 50 µl. The temperature profile was 95°C for 3 min followed by 18 cycles of amplification (95°C for 20 s, 57°C for 4 min and 72°C for 20 s on a Biorad CFX96). The samples were diluted 5 times in water. The expression of all genes was measured in preamplified and non-preamplified samples. The average difference between preamplified and non-preamplified samples and the standard deviations of the differences were calculated. The same setup was then used for the preamplification of single cells.

### High Throughput qPCR

The sample reaction mixture had a volume of 5 µl and contained 2.4 µl of diluted preamplified cDNA, 0.25 µl of DNA Binding Dye Sample Loading Reagent (Fluidigm), 2.5 µl SsoFast EvaGreen Supermix (Biorad), and 0.01 µl ROX (Invitrogen). The primer reaction mixture had a final volume of 5 µl and contained 2.5 µl Assay Loading Reagent (Fluidigm) and 2.5 µl of a mix of reverse and forward primers, corresponding to a final concentration of 4 µM. The chip was first primed with an oil solution in the NanoFlex™ 4-IFC Controller (Fluidigm) to fill the control wells of the dynamic array. The reaction mixture (5 µl) was loaded into each sample well, and 5 µl of the primer reaction mixtures was loaded into each assay well of the 48×48 dynamic array. The dynamic array was then placed in the NanoFlex™ 4-IFC Controller for automatic loading and mixing. After 55 min the dynamic array was transformed to the BioMark qPCR platform (Fluidigm). The cycling program was 3 min at 95°C for activation, followed by 40 cycles of denaturation at 95°C for 5 s, annealing at 60°C for 15 s, and elongation at 72°C for 20 s. After PCR, melting curves were collected between 60°C and 95°C with 0.5°C increments.

### Data Pre-processing

qPCR data were pre-processed for expression analysis using GenEx (MultiD, version 5.3) as follows: Cq values registered from amplifications that generated melting curves with aberrant Tm were removed, Cq values larger than 26 were replaced with 26, and Cq values with products giving rise to a double peak in melting curves (corresponding to a mixture of expected and aberrant PCR products) were replaced with 26. All missing data, for each gene separately, were then replaced with the highest Cq +2, effectively assigning a concentration of 25% of the lowest measured concentration to the off-scale values. The Cq data were, for each gene separately, converted into relative quantities expressed relative to the sample with the lowest expression (maximum Cq) and finally converted to a logarithmic scale with base 2. The data were not normalized to any reference genes because of the large variation of all transcript levels among individual cells [33]; with this pre-processing, the levels are expressed per cell.

### Analysis

Basic statistics were calculated. The non-parametric Mann-Whitney test was used to compare the expression of individual genes between groups of cells. Kohonen self-organizing maps (SOM) of size 3×1, dividing the cells into three groups, were trained using GenEx with the following parameters: 0.10 learning rate, 3 neighbors and 5000 iterations. The SOM analysis was repeated eight times with identical classification of the cells in six of the repeats. This classification of cells into three groups was substantiated with principal component analysis (PCA). The division of cells into three groups by SOM does not imply that there are three distinct subtypes of cells; the cells may also be changing their expression pattern from one state to another, and the SOM then divides the cells into different phases: those just starting to change, those that have undergone substantial change, and finally cells approaching completion. Distinct subtypes, when such are present, appear as separated clusters in PCA. For Kohonen SOM and PCA the data were mean-centered to reduce the effect of variation in the overall expression levels of the different genes. The Spearman correlation coefficients between genes were calculated for each group separately (SAS vers. 9.2) using all data, including the managed off-scale data, and then also for the truly positive expression values only. Only correlation coefficients for genes expressed in at least 50% of cells and having a p-value <0.05 were considered for further analysis. Indirect correlations between genes were established by calculating partial Spearman correlation coefficients (only genes expressed in at least 80% of the cells were considered). An interaction was considered indirect when the correlation coefficient lost significance (at 95% confidence) or was reduced to below 0.4 [28].

## Results

EGFP^+^ cells from GFAP/EGFP transgenic mice are today routinely used by several research groups [4], [35] and characterizing their gene expression profile is highly desirable. Because of the possible heterogeneity of astrocytes, single cells were collected by flow cytometry, lysed, and analyzed individually by RT-qPCR. For preamplification and further analysis we selected EGFP^+^ cells that were also positive for the astrocytic markers *Glul* and/or *Eaat2* and EGFP^+^ cells that were also positive for *Cspg4* and/or *Pdgfra*, which are markers of NG2 glia. In these cells the gene expression of the following markers was measured: glial cell markers (*Eaat1, Gfap, Gfapδ, Glul, S100b, Pdgfra, Cspg4*), developmental markers nestin and vimentin (*nes and vim*), aquaporins (*Aqp1, Aqp4, Aqp9*), subunits of glutamate ionotropic AMPA receptors (*Gria1, Gria2, Gria3, Gria4*), glutamate ionotropic NMDA receptors (*Grin1, Grin2a, Grin2b, Grin2c, Grin2d, Grin3a*), glutamate ionotropic kainate receptors (*Grik1, Grik2, Grik3, Grik4, Grik5*), glutamate metabotropic receptors (*Grm1, Grm3, Grm5*), outwardly rectifying K^+^ channels (*Kcna3, Kcna4, Kcna5*), inwardly rectifying K^+^ channels (*Kcnj2, Kcnj10, Kcnj16*), two pore domain K^+^ channels (*Kcnk1, Kcnk2, Kcnk10*) and hyperpolarization-activated cyclic nucleotide-gated channels (*Hcn1, Hcn2, Hcn3, Hcn4*), voltage-gated chloride channels (*Clcn2*), transient receptor potential channels (*Trpv4*), and *Snap25* encoding synaptosomal-associated protein SNAP-25. The markers were selected based on our previous work [5], which revealed significant differences between astrocytic subpopulations in the expression of genes coding for membrane proteins.

For the profiling of a large number of genes on the Biomark platform, it is necessary to preamplify the cDNA; in this regard, we have extensively optimized and validated the preamplification protocol. Our validation scheme is based on three samples that are profiled with and without preamplification and the measured Cq values are compared. Unbiased preamplification should result in the same ΔCq for all of the genes. The reproducibility of the preamplification is reflected by the standard deviation (SD) of the three ΔCq replicates measured for each gene. For our panel and protocol the SD was below 0.5 cycles for almost all genes. Only one gene (*Grin2d*) showed a large standard deviation (SD = 3.7 cycles) (Figure S1) and was omitted in the expression analysis. Inspecting the validation results, we did not find correlation between assay efficiency and ΔCq.

**Figure 1:**
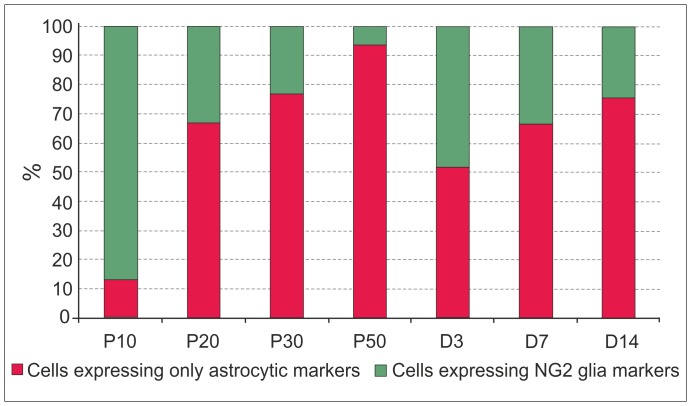
Characteristics of EGFP^+^ cells in the cortex of GFAP/EGFP mice. (**A**) Percentage of EGFP^+^ cells expressing either predominantly astrocytic or NG2 glia markers during postnatal development (P10, P20, P30, and P50) and at 3, 7 and 14 days (D3, D7 and D14) following focal cerebral ischemia. Cells expressing *Cspg4* and/or *Pdgfra* receptor are shown in green, while cells expressing only *Eaat2* and/or *Glul* are shown in red. The total numbers of the investigated cells were 137 for P10, 94 for P20, 157 for P30, 50 for P50, 148 for D3, 96 for D7 and 136 for D14.

**Figure 2:**
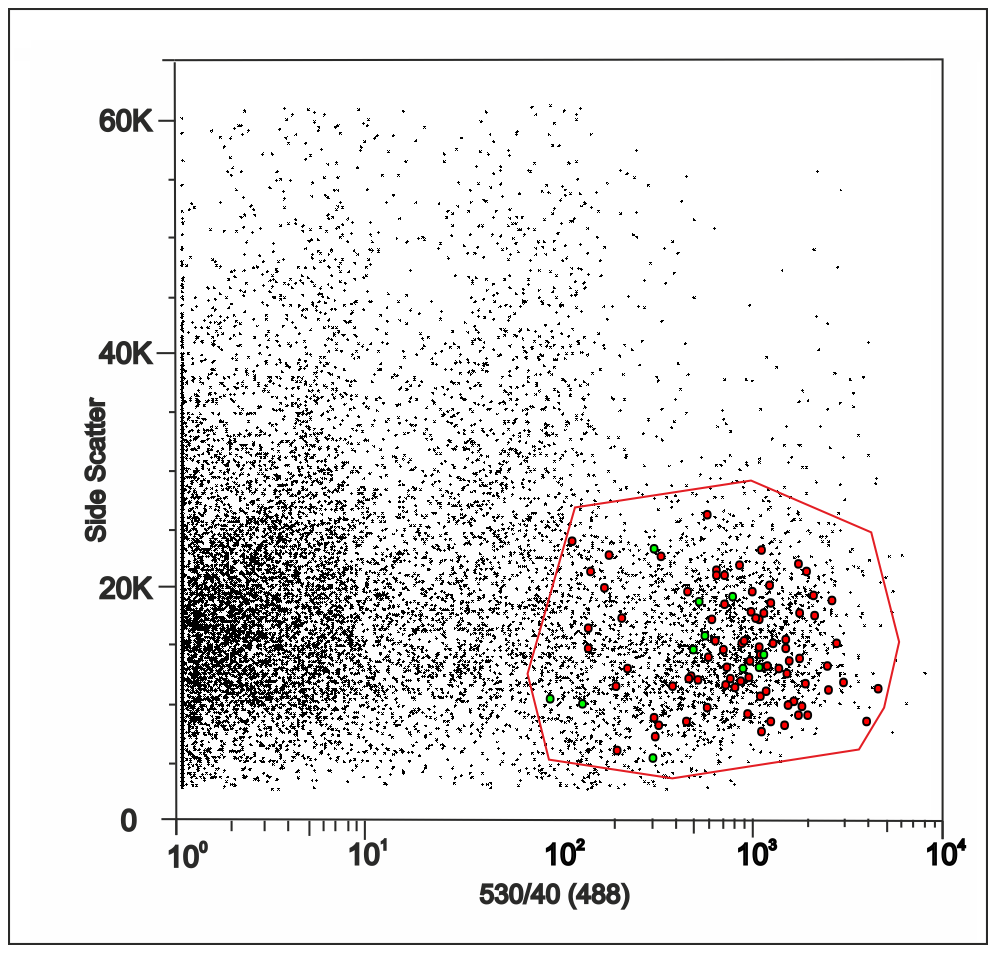
FACS analysis of EGFP^+^ cells isolated from the cortex of 20-day-old mice. EGFP^+^ cells sorted based on EGFP intensity (x axis) and side scatter intensity reflecting cell size (y axis). During the selection of cells by FACS, their position is automatically saved in the plot, and thus it is possible to determine the expression profile of each individual cell in the plot after qPCR analysis. The red line indicates the sorter gate and each dot represents one cell. Cells positive for *Cspg4* and/or *Pdgfra* are indicated in green while those positive only for *Eaat1* and/or *Glul* are shown in red. The latter are also drawn larger for better resolution. The small black dots represent all of the cells from which we selected our samples.

All cells collected from the cortex were analysed for the expression of *Eaat2*, *Glul*, *Cspg4*, and *Pdgfra* to estimate the ratio between cells that express only astrocytic markers (*Eaat2* and *Glul*) and cells that also express at least one of the NG2 glia markers (Figure 1). The number of cells that expressed only astrocytic markers increased, while the number of cells that also expressed NG2 glia markers decreased during postnatal development. After ischemic injury there was a distinct increase in the number of cells expressing NG2 glia markers 3 days after MCAO, but their number decreased again at later post-ischemic stages. In neither system did the expression profile depend on the EGFP intensity of the cells or on their size. Notably, cells expressing NG2 glia markers were distributed across the entire sorter gate (Figure 2, green dots). For single cell qPCR expression profiling 64 cells were collected from P10, 60 cells from P20, 89 cells from P30, 50 cells from P50, 90 cells from D3, 74 cells from D7, and 78 cells from D14. The cDNA produced from these cells was preamplified and measured on the Biomark platform.

### Characteristics of EGFP^+^ Cells Collected from Different Stages of Postnatal Development

**Figure 3:**
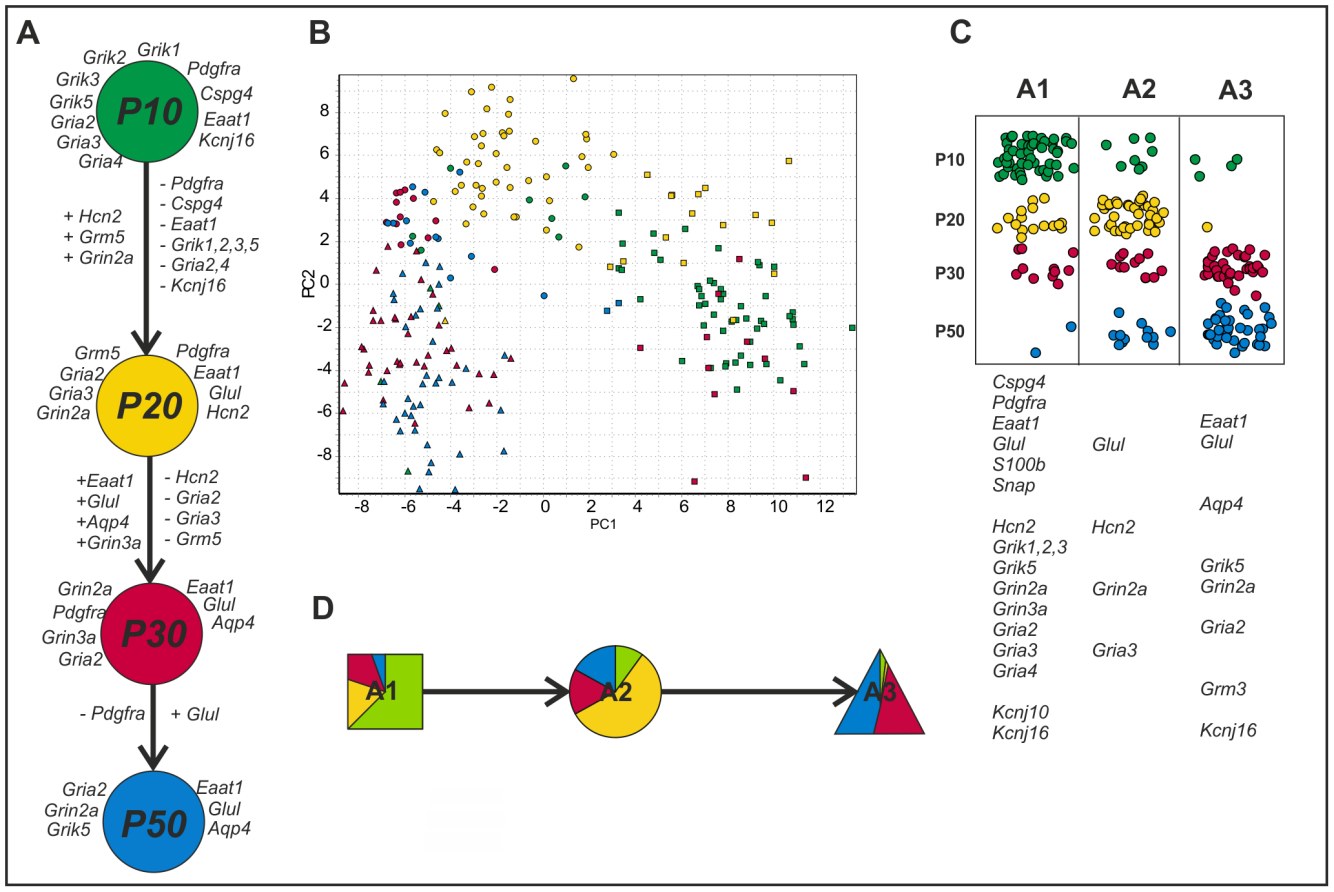
Gene expression profiling of cortical EGFP^+^ cells during postnatal development. (**A**) Changes in the gene expression of highly expressed (log_2_ relative average expression above 2.3 from all data) astrocytic/NG2 glia markers and membrane proteins at P10 (green), P20 (yellow), P30 (red), and P50 (blue). The listed genes show at least 2-fold up (+) or 2-fold down-regulation (−) between sequential developmental stages. (**B**) PCA clustering of cells from all development stages (data mean-centered along genes). The stages are indicated in color (P10 green; P20 yellow; P30 in red, and P50 in blue), and the three groups identified by SOM are indicated by symbols (squares for A1; circles for A2, and triangles for A3). (**C**) The incidence of cells from the three subpopulations at different postnatal developmental stages. The genes under the table indicate highly expressed genes (log2 relative average expression above 1.8 and at least 40% positive cells) in particular subpopulations. (**D**) The distribution of developmental stages in the subpopulations.

The majority of EGFP^+^ cells isolated from P10 mice expressed *Cspg4* and/or *Pdgfra* (87%) and *S100b* (80%). More than 70% of these cells also expressed *Eaat1*, *Grik1-3*, *5*, *Gria2-4* and *Kcnj16* (Figure 3A). Generally, cells at P10 were transcriptionally very active (Table 1). The expression profile of cells collected at P20 was different. *Cspg4* and/or *Pdgfra* were expressed in only 32% of the cells, and the expressions of *Kcnj16*, *Eaat1*, *Grik1*, *Grik3*, *Grik5*, *Gria2*, and *Gria4* were also reduced. *Gria3* expression remained high and the expression of *Hcn2*, *Grm5* and *Grin2a* increased. Cells at P30 and P50 displayed very similar expression patterns (Figure 3B). The expression of most genes was lower than at the earlier stages; only *Eaat1*, *Glul*, *Grin2a*, *Gria2* and *Aqp4* showed high expression. The fraction of cells positive for *Pdgfra* and/or *Cspg4* decreased to only 22% and 6% at P30 and P50, respectively.

**Table 1:** Statistics describing gene expression in different postnatal developmental stages or post-ischemic stages.

	Percentage of positive cells							Average relative expression log_2_						
Gene	P10	P20	P30	P50	D3	D7	D14	P10	P20	P30	P50	D3	D7	D14
**All cells (count)**	64	60	79	50	90	74	78							
***Cspg4***	77%	32%	46%	4%	49%	35%	38%	3.63	1.45	2.22	0.10	1.99	1.45	1.63
***Pdgfra***	78%	40%	51%	4%	49%	35%	42%	6.32	2.38	3.37	0.25	3.03	2.36	2.71
***Eaat1***	83%	65%	92%	90%	88%	80%	87%	4.21	2.35	5.41	6.00	4.48	4.84	5.81
***Glul***	63%	58%	92%	92%	76%	81%	92%	2.16	2.80	4.50	5.79	3.61	5.91	5.84
***Gfap***	8%	13%	27%	8%	40%	57%	40%	0.29	0.47	1.15	0.35	1.55	4.14	1.95
***Gfap D***	19%	20%	44%	32%	47%	64%	40%	0.40	0.55	1.27	0.75	1.66	3.75	1.40
***S100b***	80%	48%	62%	64%	72%	85%	82%	2.05	1.37	1.87	1.54	2.09	3.81	2.28
***nestin***	11%	3%	11%	0%	23%	16%	10%	0.24	0.11	0.46	0.00	0.92	0.68	0.45
***Vim***	31%	23%	30%	18%	60%	82%	55%	0.79	0.69	0.91	0.45	3.33	5.91	1.89
***Snap25***	61%	25%	35%	26%	27%	26%	77%	1.93	0.82	1.53	1.15	0.82	0.86	4.02
***Aqp1***	2%	50%	0%	0%	1%	4%	79%	0.03	1.13	0.00	0.00	0.02	0.13	4.89
***Aqp4***	33%	25%	65%	72%	48%	70%	59%	1.03	0.70	2.96	2.81	1.38	4.15	2.78
***Aqp9***	9%	40%	5%	2%	0%	8%	91%	0.24	1.26	0.22	0.04	0.00	0.23	6.77
***Clcn2***	27%	27%	22%	28%	33%	28%	31%	0.76	0.86	0.88	1.27	0.96	1.50	1.30
***Hcn1***	19%	43%	29%	12%	0%	22%	96%	0.46	2.21	1.24	0.48	0.00	0.94	8.81
***Hcn2***	59%	95%	0%	24%	43%	55%	95%	1.52	5.05	0.00	0.61	1.38	3.30	8.78
***Hcn3***	31%	62%	28%	10%	22%	34%	88%	0.65	2.08	0.74	0.26	0.51	0.84	6.55
***Hcn4***	67%	43%	84%	72%	24%	80%	27%	1.40	1.23	1.88	1.46	0.52	1.69	1.85
***Trpv4***	38%	17%	34%	28%	39%	34%	35%	1.17	0.40	1.16	0.81	1.13	1.27	1.25
***Grik1***	67%	30%	44%	14%	40%	47%	63%	2.37	0.91	1.70	0.43	1.28	1.89	2.26
***Grik2***	75%	58%	38%	30%	42%	61%	47%	2.67	1.65	1.39	0.90	1.31	2.87	1.52
***Grik3***	70%	32%	48%	8%	44%	31%	33%	2.54	1.11	2.17	0.21	1.62	1.28	1.25
***Grik4***	45%	15%	22%	4%	19%	30%	14%	1.14	0.42	0.76	0.08	0.53	1.12	0.37
***Grik5***	86%	47%	54%	72%	77%	68%	69%	3.45	1.42	2.14	2.38	3.26	3.39	3.07
***Grin3a***	67%	32%	61%	10%	43%	35%	51%	2.27	0.90	2.46	0.25	1.25	1.36	1.58
***Gria1***	41%	12%	16%	8%	18%	16%	28%	0.87	0.28	0.37	0.22	0.44	0.63	0.72
***Gria2***	91%	62%	52%	74%	76%	66%	77%	5.22	2.45	2.60	3.54	3.19	3.25	4.08
***Gria4***	75%	53%	44%	24%	44%	54%	42%	2.67	1.45	1.71	0.83	1.59	2.54	1.54
***Grin1***	13%	32%	39%	18%	28%	50%	50%	0.27	0.69	1.03	0.47	0.63	1.26	1.30
***Grin2a***	47%	77%	49%	48%	46%	54%	97%	1.67	3.20	2.45	2.58	1.71	3.11	8.56
***Grin2b***	61%	33%	33%	30%	44%	57%	59%	1.82	0.90	1.30	1.24	1.37	2.60	2.19
***Grin2c***	13%	8%	23%	48%	28%	30%	35%	0.29	0.17	0.57	1.55	0.79	0.96	0.94
***Grin2d***	2%	15%	14%	4%	8%	4%	64%	0.03	0.42	0.55	0.17	0.20	0.11	3.05
***Gria3***	84%	87%	41%	12%	53%	49%	100%	4.23	4.55	2.21	0.58	2.29	2.46	9.49
***Grm1***	30%	18%	27%	32%	37%	31%	31%	0.89	0.64	1.01	1.18	1.20	1.24	1.34
***Grm3***	20%	27%	33%	62%	44%	61%	41%	0.60	1.01	1.35	2.39	1.43	2.80	1.89
***Grm5***	38%	82%	29%	0%	30%	27%	92%	0.77	2.45	0.77	0.00	0.71	0.64	7.18
***Kcna3***	25%	67%	18%	14%	23%	27%	91%	0.52	1.47	0.33	0.31	0.52	0.68	6.00
***Kcna4***	33%	45%	38%	22%	19%	53%	37%	0.99	1.69	1.58	0.73	0.60	2.36	1.48
***Kcna5***	25%	30%	42%	22%	41%	64%	38%	0.79	0.89	1.58	0.64	1.29	2.83	1.33
***Kcnj10***	95%	45%	72%	74%	79%	82%	88%	2.05	1.12	1.33	1.48	1.92	3.04	1.96
***Kcnj16***	66%	30%	54%	52%	60%	55%	58%	2.37	0.96	2.11	2.15	2.25	2.64	2.38
***Kcnj2***	17%	17%	19%	14%	23%	49%	29%	0.51	0.56	0.73	0.44	0.81	2.12	0.96
***Kcnk1***	22%	15%	30%	24%	26%	11%	28%	0.53	0.44	1.10	0.65	0.62	0.43	0.94
***Kcnk10***	67%	25%	33%	20%	44%	50%	32%	1.54	0.55	0.78	0.48	1.02	1.23	0.79
***Kcnk2***	34%	17%	23%	4%	31%	12%	41%	1.09	0.41	0.80	0.16	0.87	0.42	1.35

Cells collected between P10 and P50 were divided by SOM into 3 groups based on their expression profiles (Figure 3C). This SOM classification was fully reproducible - repeated independent classifications predicted the same groups. This shows that the collection of cells is not homogeneous. SOM itself cannot tell if there are distinctly different groups or gradual changes, but the cells can be divided reproducibly into three subpopulations. The spatial separation between the clusters in the PCA suggests that the subpopulations are distinct (Figure 3B). Dividing the cells into more than three groups with SOM did not reveal any additional differences and was not supported by the PCA. We compared the average expression of the individual genes in the three subpopulations, which we henceforth refer to as A1, A2 and A3 (Table S2). Subpopulation A1 was characterized by the high expression of *Pdgfra*, *Cspg4*, *Grik1-3*, *Grik5*, *Gria2-4*, *Grin2a*, *Grin3a*, *Kcnj16*, *Eaat1*, *Glul*, *S100b*, *Snap25* and *Kcnj10* (Figure 3C) and was comprised mainly of P10 cells (composition: 64%, 19%, 13% and 2% of P10, P20, P30 and P50, respectively; Figure 3D). Subpopulation A2 was characterized by the low expression of most genes, including *Eaat1*, *Cspg4*, *Pdgfra*, *Grik5*, *Gria2* and *Grin3a*; only *Glul*, *Hcn2*, *Gria3* and *Grin2a* were highly expressed. Subpopulation A2 was comprised mainly of P20 cells (57%); cells from the other stages (P10, P30 and P50) were present at similarly low levels (Figure 3D). In addition to the classical astrocyte markers *Eaat1*, *Glul*, Aqp4, subpopulation A3 also expressed *Grik5*, *Gria2-3*, *Grin2a*, *Grm3* and *Kcnj16* at high levels and was comprised mainly of P30 and P50 cells (46% of each).

The relation between the expressions of different genes among the individual cells can be evaluated by calculating correlation coefficients. The calculation can include all cells, including those that did not express the genes of interest, or only those cells that had measurable expression. When all cells are considered, the dominant effect is whether a cell expresses both genes or not, while when cells with missing data are filtered out, the correlation coefficient is dominated by the expression levels of genes (Table S3, S4 and S5, Figure S2A–C). Although 95% of all cells in subpopulation A1 expressed *Pdgfra* as well as *Cspg4*, the correlation between their expressions was weak (r = 0.44), which means that they are expressed independently of each other in individual cells. We hypothesize that their expression might be regulated by different transcriptional factors and/or that such a weak correlation between *Pdgfra* and *Cspg4* expression might reflect the different stages of astrocyte maturation even within the A1 subpopulation, which comprises EGFP^+^ cells from P10 and P20. The correlation between the expression of *Gria2* and *Cspg4* was negligible, while *Gria2* expression correlated with *Pdgfra* (r = 0.60) (Table S3). In subpopulation A3, correlated expression was found between *Glul* and *Eaat1* (r = 0.59) when considering only cells with valid expression data (74 cells) and between *Eaat1* and *Gria2* (r = 0.73). Notably, the correlation of *Eaat1* with *Glul* and of *Eaat1* with *Gria2* in subpopulations A1 and A2 was much lower, thus the increasing correlation between *Glul* and *Eaat1* clearly mirrors the maturation process of astrocytes.

**Figure 4:**
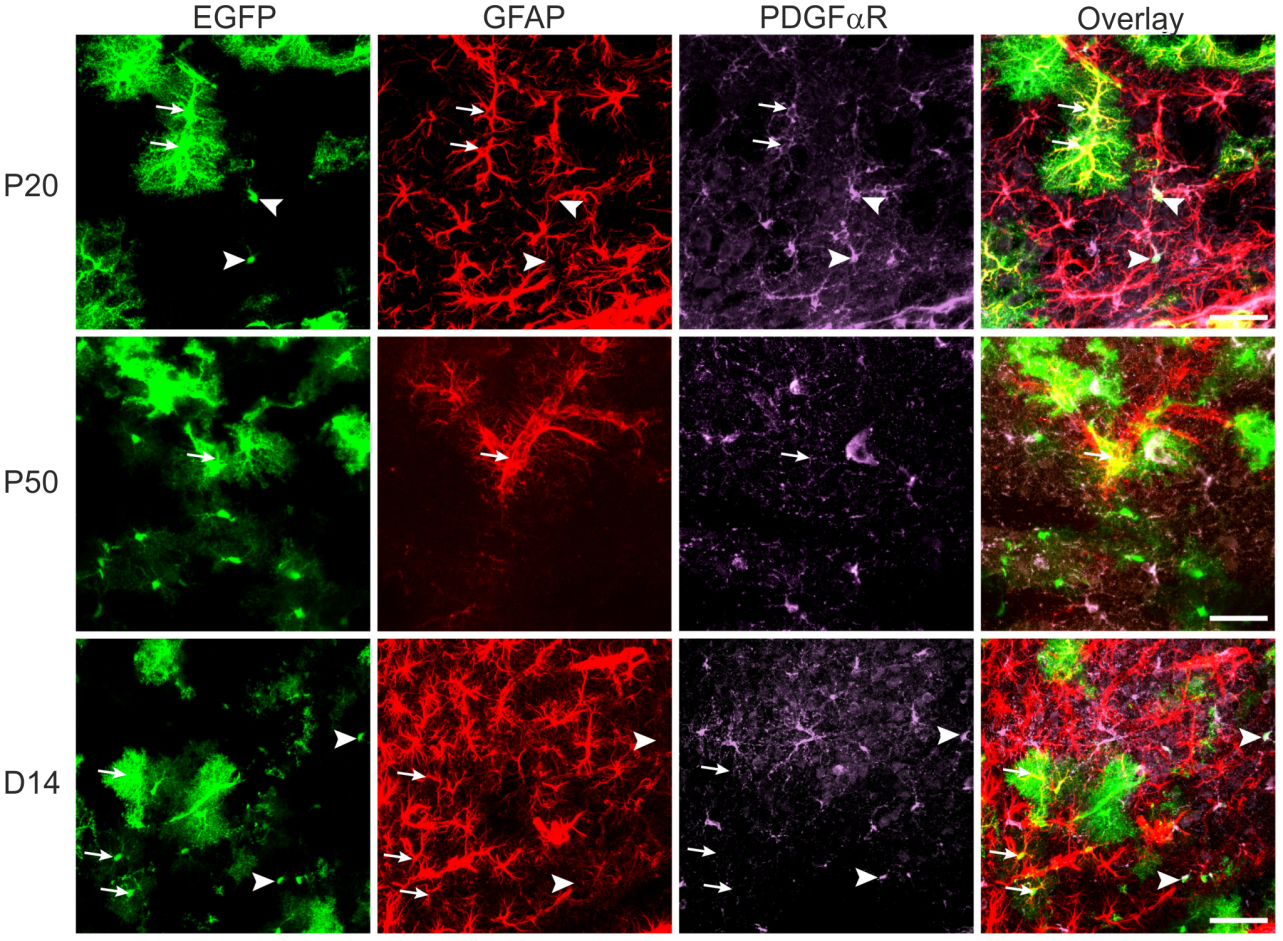
Immunohistochemical analysis of EGFP^+^ cells. P20, P50, and D14 cells expressing EGFP (green), GFAP (red), and PDGFαR (violet). Arrowheads indicate cells co-expressing EGFP and PDGFαR, and arrows indicate cells positive for EGFP and GFAP. Note, at P20 there are cells positive for all three markers. Scale bars, 50 µm.

Interestingly, gene expression profiling revealed that a large number of EGFP^+^ cells express *Cspg4* as well as *Pdgfra* at P10, while at P50 we detected only a negligible number of EGFP^+^ cells that co-expressed these markers (Figure 1). Furthermore, we also detected the co-expression of *Gfap* and *Cspg4/Pdgfra* in most of the cells at P10; this co-expression gradually decreased at P20 and P30, and at P50 there was no co-expression detected. In order to confirm this observation on the protein level, we carried out immunohistochemical analyses, which revealed the co-expression of GFAP and PDGFαR or of GFAP and NG2 in EGFP^+^ cells only at P10 and P20, but not at P50 (Figure 4, Figure S3).

### Expression Profiling of Reactive Glia Following Focal Cerebral Ischemia

**Figure 5:**
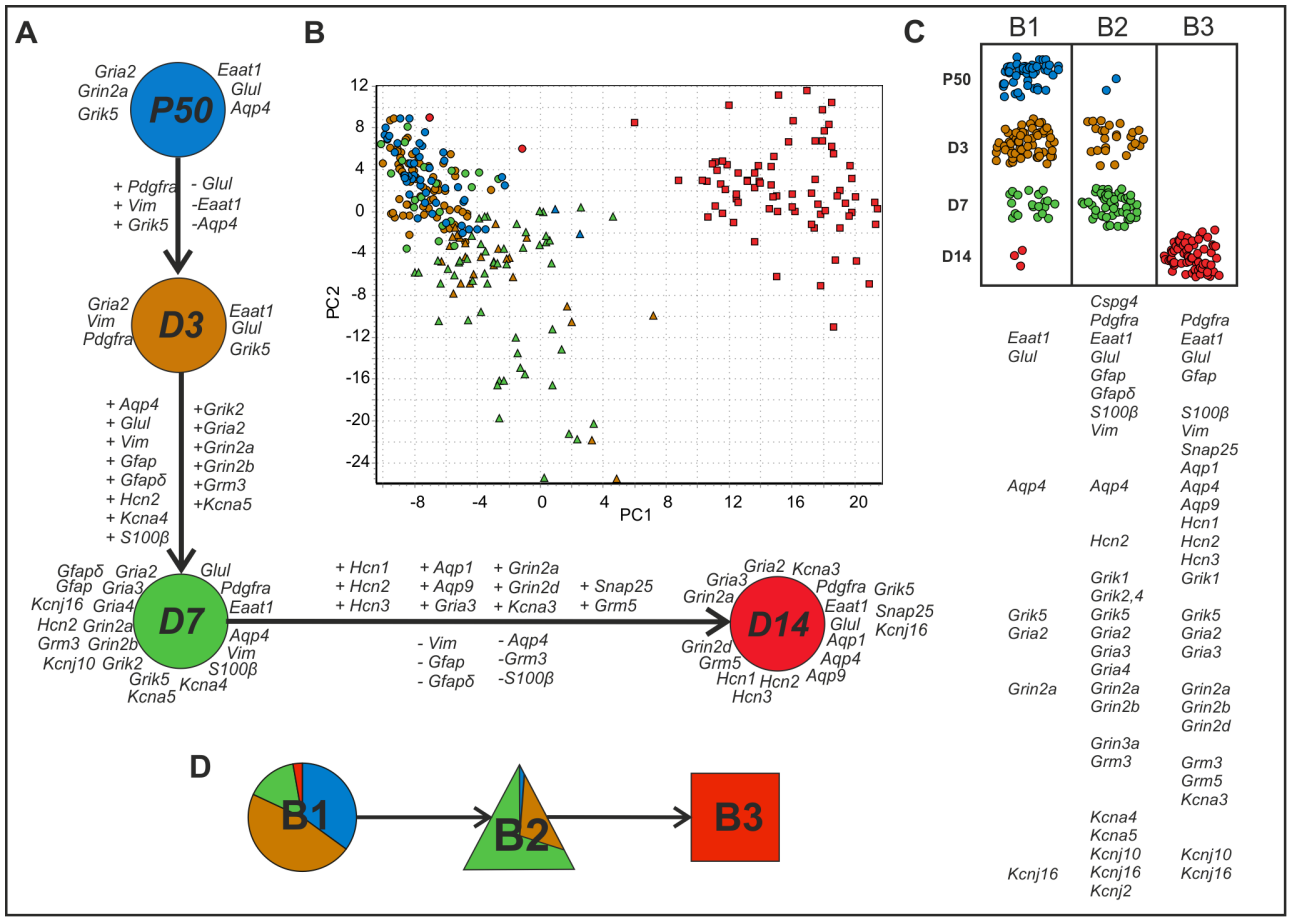
Change in the gene expression of EGFP^+^ cells after MCAO. (**A**) Changes in the gene expression of highly expressed (log_2_ relative average expression above 2.3 from all data) astrocytic/NG2 glia markers and membrane proteins at P50 (blue), D3 (orange), D7 (green), and D14 (red). The listed genes show at least 2-fold up (+) or 2-fold downregulation (−) between sequential stages. (**B**) PCA clustering of cells from all post-ischemic stages and P50 (data mean-centered along genes). The stages are indicated in color (P50 blue, D3 orange, D7 green, and D14 red), and the three groups identified by SOM are indicated by symbols (squares for B3; circles for B1 and triangles for B2). (**C**) The incidence of cells from the three subpopulations at post-ischemic stages and P50. The genes under the table indicate highly expressed genes (log2 relative average expression above 1.8 and at least 40% positive cells) in particular subpopulations. (**D**) The distribution of control and post-ischemic stages in individual subpopulations.

Three days after ischemia the expression profiles of the cells had changed. Approximately 50% of all EGFP^+^ cells began to express *Pdgfra*, *Vim*, and *Grik5*, while *Aqp4*, *Glul* and *Eaat1* expression was decreased. Seven days after ischemia the cells were transcriptionally more active with particularly high expression of *Pdgfra*, *Vim*, *S100b*, *Eaat1, Glul*, *Gria2-4*, *Grin2a*, *Grin2b*, *Grik2*, *Grik5*, potassium channels *Kcna4*, *Kcna5*, *Kcnj10*, *Kcnj16*, *Aqp4* and *Hcn2*. Furthermore, the cells also began to express *Gfap* and *Gfapδ*. At this time, the expression of *Glul* returned to the basal level (P50, Figure 5A). Fourteen days after ischemia, the difference in expression compared to control (P50) was the largest. The expression of *Vim*, *Gfap*, *Gfapδ*, *Aqp4*, *S100b* and *Grm3* had dramatically decreased, while the expression of *Hcn1, Hcn2, Hcn3*, *Aqp1*, *Aqp9, Grm5*, *Gria3*, *Grin2a*, *Grin2d*, *Snap25* and *Kcna3* had markedly increased. At all post-ischemic stages, we found some cells expressing *Cspg4* and *Pdgfra*.

All cells (P50, D3, D7 and D14) were divided by SOM into 3 subpopulations and confirmed by PCA to have different expression profiles (Figure 5B). We will refer to these subpopulations as B1, B2 and B3. To characterize these subpopulations, we compared the expressions of the genes individually in histograms and by conventional univariate statistics. Almost all cells from P50 were assigned to subpopulation B1 (48 out of 50) (Figure 5C), and they comprised 35% of the B1 cells. 47% of B1 cells were from D3, 15% from D7, and only 3 B1 cells were from D14 (Figure 5D). The B1 subpopulation displayed the lowest overall transcriptional activity; high expression was observed only for *Eaat1*, *Glul*, *Aqp4* and *Gria2*. Subpopulation B2 was more transcriptionally active with the high expression of genes encoding glutamate ionotropic receptors, K^+^ channels, *Grm3, Aqp4*, *Hcn2, Gfap, Gfapδ, Glul, Eaat1, S100b, Vim, Cspg4,* and *Pdgfra*. Most B2 cells were from D7 (66%), 31% were from D3 and only 2 cells were from P50. Notably, the two P50 cells also expressed *Pdgfra* and *Cspg4*. Subpopulation B3 was composed of only D14 cells. The very high expression of many genes makes this group unique, and there is no overlap in the PCA between B3 and the other subpopulations. Clearly, the B3 subpopulation is distinct. We also noted that the EGFP fluorescence of these cells was much higher compared to that of cells classified as B1 and B2.

Generally, astrocytes isolated from post-ischemic stages (D3, D7 and D14) showed much higher gene expression than those collected from the postnatal developmental stages. Within each subpopulation B1-B3 we found many significant correlations. The strongest are listed in Tables S6, S7 and S8 and visualized in Figure S2D–F. Interestingly, the expression of *Pdgfra* and of *Cspg4* were strongly correlated when cells with non-measurable expression (r = 0.89, 0.71 and 0.87 in B1, B2 and B3) were included, but the correlation was lost when only cells with measurable expression were considered. The expressions of *Eaat1* and *Glul* in B1 and B3 were correlated (r = 0.66 and 0.79), and a correlation of *Eaat1* and *Aqp4* was found in B1, B2 and B3 (r = 0.43, 0.69, 0.81). A strong correlation was also found between the expressions of *Gfap* and *Gfapδ* in B1 and B2 (r = 0.76 and 0.87) and between the expressions of *Aqp1* and *Aqp9* in B3 (r = 0.78). The expressions of the latter two genes also correlated with those of *Hcn1, Hcn2, Hcn3, Hcn4*, *Grin2a*, *Grin2d, Gria3, Grm5, Kcna3, and Kcnk2*. In fact, the expressions of almost all of these genes were strongly correlated on the single cell level in B3 (r close to or larger than 0.7, Figure S4A). Such a strong correlation between the above-listed genes suggests significant alterations in the membrane properties of reactive astrocytes at D14, namely the appearance of *Hcn1-4,* which so far have been described only in neurons [36]. On the other hand, a significant negative correlation was found between the expressions of *Cspg4* or *Pdgfra* and *Glul, Aqp9, Hcn1, Grin2a* and *Gria3* (r<-0.55). This clearly demonstrates that astrocytes from D14 (B3 subpopulation) lose the expression of *Cspg4* and *Pdgfra* while they change into reactive astrocytes comprising a permanent glial scar.

The calculated partial Spearman correlation coefficients surprisingly indicated that there was no direct interaction between *Aqp9* and *Aqp1* (Figure S4B), but the observed correlation can be accounted for as being mediated via *Hcn2* or *Kcna3*. Further, *Aqp9* does not mediate any interaction between *Hcn1, Hcn2, Hcn3, Gria3, Grm5* and *Aqp1*. Out of the total 21 possible direct interactions, *Gria3* and *Hcn2* were involved in 10 (Figure S4C and S2D).

**Figure 6:**
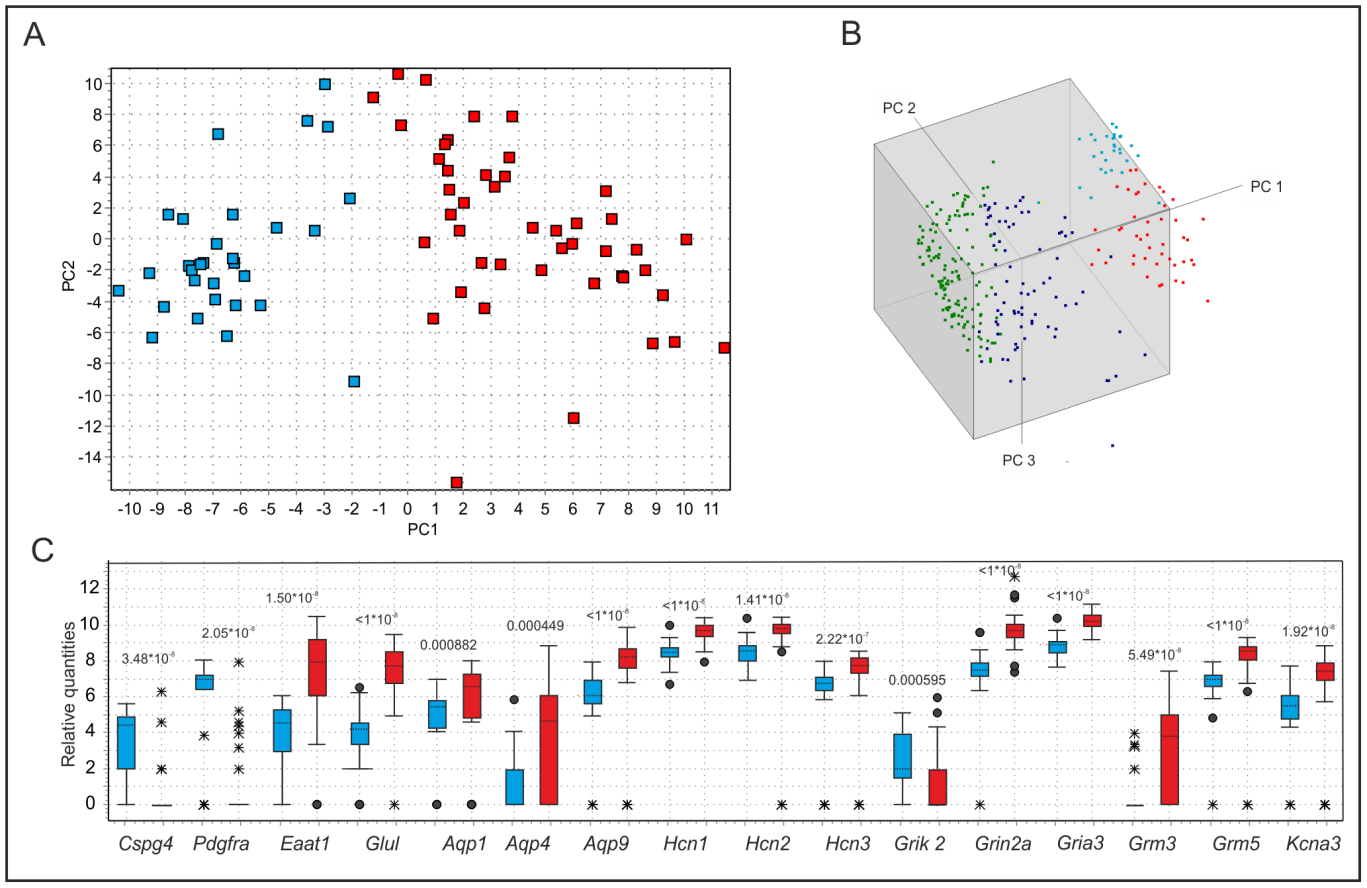
Classification of reactive glia (B3) into two subgroups. (A) PCA scatter plot of the cells in subpopulation B3 (reactive glia). Colors indicate the two groups (B3A red and B3B blue) obtained by decomposing the data by SOM analysis (data are mean-centered). (B) Three-dimensional PCA scatter plot of all post-ischemic cells and P50 reflecting the normal state for comparison - B1 (green), B2 (dark blue), B3A (red) and B3B (light blue). PC3 separates B3A from B3B. The first PC accounts for 32%, the second 14% and the third 8%, of the total variance in the data. (C) Box plots showing genes differentially expressed in B3A (red) and B3B (blue). The box indicates the median value and the 25th and 75th percentiles, the whiskers are 1.5× the distance of the 25th and 75th percentiles, the circles indicate outliers and the crosses extreme outliers. p-values (Mann-Whitney) are indicated for each gene.

Further classification of the reactive glial cells in subpopulation B3 using SOM revealed that it is not a homogenous group; rather there are two subgroups, which we refer to as B3A and B3B. Their existence was clearly confirmed by PCA (Figure 6A). Viewing the data in a 3-dimensional PC1 vs. PC2 vs. PC3 space shows that the B3A/B3B subpopulations separate along PC3 (Figure 6B). Hence, the genes distinguishing B3A from B3B are those with high weights in PC3, thus being different from the genes distinguishing B3 from B1/B2, which are those with high weights in PC1 and PC2. The expression of genes in the B3A and B3B subgroups were compared using the non-parametric Mann-Whitney test, and those with significant p-values after Bonferroni correction for multiple testing (p<0.00109) are shown in Figure 6C. The most important differences between the B3A and B3B subgroups are the down-regulation of *Pdgfra, Cspg4* and *Grik2* and the up-regulation of *Eaat1, Glul, Aqp1, Aqp4, Aqp9, Hcn1, Hcn2, Hcn3, Grin2a, Gria3, Grm3, Grm5* and *Kcna3* (Figure 6C).

Similarly as in the early stages of development, post-ischemic astrocytes also co-expressed *Gfap* and *Cspg4/Pdgfra* (approximately 40%); however, on the protein level we did not detect such co-expression (Figure 4).

## Discussion

We show that cortical EGFP^+^ cells collected during postnatal development as well as cells collected after focal cerebral ischemia are highly heterogeneous with respect to their gene expression profiles. Our findings demonstrate that the issue of astrocyte heterogeneity is complicated, but can be approached with single cell expression profiling, which reveals and distinguishes cell subpopulations. With single cell resolution we can identify rare cells, an approach impossible in classical bulk measurements [37]. Furthermore, single cell profiling avoids the ambiguity of normalization [38].

### Astrocyte Heterogeneity during Postnatal Development

We have shown that during postnatal development, the number of EGFP^+^ cells expressing the astrocytic markers *Glul* and/or *Eaat1* increases towards adulthood, while the number of cells expressing *Cspg4* and/or *Pdgfra* is reduced. Similarly to previous findings, we show that in addition to *Glul*, the expression of *Eaat1*, *Aqp4* and *Grin2a* is induced during development [39]. This is an opposite tendency to that reported by Stanimirovic and colleagues [40], but their experiments were carried out *in vitro*, in a purely astrocytic culture, where glutamate uptake and glutamine synthesis might be compromised. We also found a substantial decrease in *Grik2, Grik3, Gria2, Gria3* and *Gria4* expression during development, which is in agreement with previous data demonstrating the expression of kainate receptors in glial precursor cells, while mature astrocytes show an NMDA-specific responses [41], [42]. Similar decline in *Grik1-5* expression during development was described recently by Cahoy and colleagues (Cahoy et al., 2008). In agreement with the findings of Matthias and colleagues [43], we found that EGFP^+^ cells that express *Cspg4* and/or *Pdgfra* also express *Gria2-4* at high levels, while the low expression of *Gria2-4* is observed in EGFP^+^ astrocytes that do not express markers of polydendrocytes. These authors also claimed that EGFP^+^ cells co-express glutamate receptors and transporters, which is supported by our single cell data. As shown in Table 1, we found the co-expression of glutamate receptors and glutamate transporters in individual EGFP^+^ cells. Among cells collected at the postnatal developmental stages P10-P50, we distinguish 3 subpopulations here referred to as A1, A2 and A3 (Figure 3). A3 cells exhibit features previously described in mature astrocytes with very high expression of *Eaat1, Glul* and *Aqp4* [23]. Cells in A1 also express *Eaat1*, but they also have high levels of *Cspg4* and *Pdgfra*, which are traditionally associated with NG2 glia [44]. Nevertheless, *Cspg4* expression in P1-P17 astrocytes was already described elsewhere (Cahoy et al., 2008). Subpopulation A2 is the most unique with high expression of *Glul, Hcn2, Gria3*, and *Grin2a*, while the transcriptional activity of the other studied genes is rather low. Based primarily on the elevated expression of *Glul* and the low *Gria3* levels, we hypothesize that A2 is an intermediate stage between A1 and A3 (Table S2). This hypothesis is also supported by the prevalence of A2 cells at P20. It is still a matter of debate whether astrocytes can be derived from CSPG4- and/or PDGFαR-positive cells; some recent evidence suggests this possibility following CNS injuries [9], [12]. Interestingly, the A1 subpopulation, mostly comprising cells from P10–20, is characterized by the increased expression of subunits of ionotropic AMPA/kainate receptors, such as *Grik1-3, Grik5, or Gria2-4,* which makes these cells sensitive to AMPA/kainate receptor–mediated glutamate injury. The presence of *Gria2* encoding GluA2 subunits suggests that AMPA receptors in the A1 subpopulation might be impermeable for Ca^2+^. The subpopulations A3 and B1, which mainly represent mature astrocytes at P30-P50, show besides the expression of classical markers such as *Eaat1, Glul* and *Aqp4*, also the dominant expression of *Gria2*, suggesting AMPA receptor impermeability to Ca^2+^. Moreover, *Kcnj10* and *Kcnj16*, encoding inwardly rectifying Kir4.1 and Kir5.1 channels, are both highly expressed in immature cortical glia, while in mature astrocytes *Kcnj16* prevails. Since heteromeric Kir4.1/5.1 and homomeric Kir4.1 have distinct ion channel properties, such differences in the expression of K^+^ channels that participate in K^+^ uptake might result in the diverse ability of astrocytes to take up/distribute high extracellular K^+^ during increased neuronal activity or in pathological states, such as ischemia or trauma. Notably, even though mature/immature astrocytes have been shown to display different K^+^ current profiles during development in the rat hippocampal CA1 region [45], such as outward rectifying, variably rectifying and passive current patterns, we found no significant differences in the transcription activities of inwardly and outwardly rectifying K^+^ channels or K2P channels between the A1-A3 subpopulations. We found the expression of *Kcnj10* (encoding the Kir4.1 channel) in the EGFP^+^ cells to be rather constant, while higher levels of *Kcnj16* (encoding Kir5.1) transcripts were found in cells from subpopulations A1 and A3. We did not see any correlation between *Aqp4* and *Kcnj10* expression on the cellular level in any of the subpopulations in agreement with previous data showing the lack of any direct interaction between Kir4.1 and AQP4 [46], [47] and their different regulations of expression [48]. In agreement with recent findings [25], [49] we showed that EGFP^+^ mature cortical astrocytes from P30 and P50 (comprising the B3 subpopulation) express low levels of *Grm5,* as a result of a significant decrease in *Grm5* expression towards astrocytic maturation, and a high level of *Grm3.* Nedergaard and colleagues suggested that mGluR5 activity underlies glutamate–induced Ca^2+^ signalling only in immature postnatal astrocytes, while in mature astrocytes other transmitters, such as endocannabinoids, purines, norepinephrine, and acetylcholine, as well as changes in extracellular Ca^2+^, may trigger astrocytic Ca^2+^ signalling. The activation of mGluR3, a Gi/Go receptor negatively coupled to adenylate cyclase, probably does not participate in glutamate-induced Ca^2+^ signalling as its activation did not trigger a Ca^2+^ increase in adult astrocytes [49]. Since the activation of mGluR3 inhibits cyclic adenosine monophosphate (cAMP) formation, it might participate in the regulation of processes such as astrocyte proliferation, the expression of glutamate receptor GLAST or GFAP expression [50], [51].

### Gene Expression in Post-ischemic Astrocytes

After CNS injury, such as trauma or ischemia, or in CNS neurodegenerative disorders, the properties of astrocytes change; the cells become reactive [52], [53]. This is in line with our finding that the gene expression profile of astrocytes alters following focal ischemia. Particularly affected is the expression of *Gfap, Gfapδ, S100b* and *Vim*, which was also observed previously [13], [53]. Fourteen days after ischemia, *Aqp1* and *Aqp9* were up-regulated as described earlier [14], [15], and similarly to previous reports, we found upregulated *Grm5* [18], [19]. Generally, *Aqp1* and/or *Aqp9* are elevated in hypertrophied, GFAP overexpressing astrocytes in a number of pathological states, such as ischemic or traumatic brain injury, Alzheimer’s or Creutzfeldt-Jakob diseases [54]–[56]. In the present study, we detected their increased expression only in the B3 subpopulation, which comprises mostly EGFP^+^ cells 14 days after MCAO. Such a delayed increase in their expression suggests that they are probably not implicated in water movements during oedema formation/resolution, but they might play a role as metabolite channels facilitating the diffusion of glycerol and lactate, as suggested previously [57], [58]. Moreover, the ischemic brain undergoes marked remodelling, which involves the migration of neighbouring cells to the injury site, thus the increased *Aqp1* expression may reflect a migratory phenotype of reactive astrocytes as AQP1 was shown to promote cell migration in epithelial cells [59] and glial tumors [60], [61]. The B2 and B3 subpopulations also highly express AMPA receptor subunits, especially *Gria3*, which suggests that AMPA receptors may significantly contribute to Na^+^/Ca^2+^ influx into the reactive astrocytes, and together with the strong expression of metabotropic glutamate receptors (*Grm1, Grm5*) these receptors possibly mediate Ca^2+^ oscillations and glutamate release, as mentioned previously [62]. Furthermore, an increase in *Grm5* expression in reactive astrocytes might lead to the increased activity of mGluR5 and thus contribute to astrocytic apoptosis, as described recently [63]. On the other hand, an anti-apoptotic effect mediated by mGluR3 activation that leads to reduced intracellular levels of cAMP was described in cultured astrocytes after their exposure to nitric oxide [64]. In addition, Aguado and colleagues [65] demonstrated that the activation of ionotropic glutamate receptors is required for the generation of correlated astrocytic network activity, essential for neuronal development and synaptic plasticity. In mature astrocytes under physiological conditions (P50), the Ca^2+^ permeability of AMPA receptors is diminished compared to immature stages (P10) due to the predominant expression of *Gria2*. Interestingly, mRNAs encoding kainate receptor subunits (*Grik1,2,3,5*) are also significantly increased in the B2 and B3 subpopulations; however, their function in reactive astrocytes and those from immature (P10) animals has not yet been elucidated.

Marked changes were also observed in the expression of different types of ion channels, such as outwardly and inwardly rectifying K^+^ channels and chloride channels (ClC2), and moreover, Trpv4 expression almost doubled after ischemia, when comparing the B2 and B3 subpopulations to B1. Despite the fact that the expression of *Kcna3-5*, encoding the outwardly rectifying K^+^ channels Kv1.3, Kv1.4 and Kv1.5, was comparable in the A1–3 subpopulations during development, it markedly increased in the post-ischemic B2 and B3 subpopulations. Such an increase might point to the fact that astrocytes enter the cell cycle and proliferate following ischemic injury, as shown previously [66]. In addition, an increase in *Clc2* expression could suggest the participation of astrocytes in delivering Cl^−^ to the site of intensive GABAergic transmission [67], or Cl^−^ efflux can play a role in astrocyte proliferation/migration as suggested for gliomas [68]. Furthermore, enhanced *Trpv4* expression following MCAO was detected in both the B2 and B3 subpopulations, which is in agreement with our recent data demonstrating an increased expression/function of TRPV4 following hypoxic/ischemic injury of the rat hippocampus [69]. TRPV4 upregulation might originate from its role as an osmosensor as shown in astrocytes [70] or from a possible role of this channel in Ca^2+^ signalling in post-ischemic astrocytes, which may result in astrocytic cell death [71]. A transient increase in *Nes* observed in cells 3 and 7 days after MCAO might support the hypothesis that these subpopulations comprise proliferative/newly derived cells. A striking finding is the high expression of *Hcn1-4*, which encodes hyperpolarization–activated non-specific cationic channels permeable for K^+^/Na^+^_,_ so far described only in neurons. Despite the fact that their role in astrocytes is unknown, we hypothesize that they might have a role in determining the astrocyte resting membrane potential or they might interact with the glutamate release machinery as described recently for presynaptic terminals [72]. A question that still remains incompletely elucidated is the origin of reactive glia. Certain subpopulations of reactive astrocytes have been shown to arise from NG2 glia [73] or from proliferating astrocytes [74]. Based on our findings, we propose that the B2 subpopulation, which is characterized by an increase in the expression of several astrocytic markers including *Gfap, S100b, Gfapδ* and *Vim,* but also by a marked increase in *Nes, Pdgfra* and *Cspg4,* comprises astrocytes in an “intermediate stage” that increase in number as astrogliosis develops (Figure 5). We hypothesize that either NG2 glia give rise to the B2 subpopulation, which eventually develops into fully reactive astrocytes with a distinct expression profile as in subpopulation B3, or they might represent dedifferentiated mature astrocytes with increased proliferative activity. The latter possibility is supported by the fact that they display a relatively similar gene expression profile as EGFP^+^ cells of the immature cortex (Figure 3). Interestingly, B1 and B2 show some similarity (e.g., the expression of *Pdgfra, Cspg4, Grik1-3, Grik5, Gria2-4, Grin2a* and *Kcnj16*) to the A1 subpopulation observed during early postnatal development (Table S2). Using partial Spearman correlation calculations, we did not identify any master gene in B3 that would account for all of the changes observed upon astrocyte reactivity that follows ischemic injury; however, it is clear that *Gria3* and *Hcn2* are of high importance. In subpopulation B3 we identified several, previously not recognized highly expressed genes: *Hcn1-3, Grin2a, Gria2, Gria3, Grm5* and *Kcna3*. We also found the high expression of *Snap25*, which is expressed only minimally in the other subpopulations and has been suggested to be a marker of cells with exocytotic capabilities under physiological conditions [25]. Similar observations were made in rats [75], where SNAP25 was found upregulated 3 and 12 days after injury. Despite the fact that synaptosomal-associated protein SNAP-25 has not been detected in astrocytes but SNAP-23 [76], our data revealed mRNA levels coding SNAP-25 were significantly increased in cortical astrocytes 14 days post-ischemia (B3 subpopulation). As astrocytes are known to release glutamate and aspartate in response to elevated intracellular calcium levels via a vesicular release mechanism in which SNARE proteins are implicated, high levels of *Snap25* might be a sign of a significant glutamate release from reactive astrocytes. We found a positive correlation between the expressions of *Snap25, Eaat1* and *Glul* and the absence of any correlation with *Cspg4* and *Pdgfra* in B3. We also found a strong correlation between *Eaat1* and *Aqp4* expression, which is in accordance with a recent report suggesting the close relation of excitatory amino acid transporters (*Eaat*) and *Aqp4* under pathological conditions with the massive release of glutamate [14]. Although an association between AQP4 and mGluR5 has been reported [77], we found no correlation on the gene expression level. On the other hand, we did find an extremely strong correlation between *Aqp1/Aqp9* and *Grm5* levels in B3.

Heterogeneity of reactive astrocytes in the vicinity of an ischemic lesion has been described previously [22], [78], [79]. Even within the B3 subpopulation of reactive astroglia, we found evidence for two subgroups using SOM and PCA: B3A and B3B. B3A is positive for traditional astrocytic markers, while B3B has a substantially higher expression of traditional NG2 glia markers (Figure 6). *Cspg4* has been proposed as a hallmark of reactive astrocytes [16]. Even though the entire B3 population has high overall transcriptional activity, much higher than B1 and B2, there is a difference in the expression levels of many genes between the B3A and B3B subgroups. Since we isolated cells from the vicinity of an ischemic lesion, it is possible that B3 comprises cells permanently converted to reactive glia, which are in different states or at different distances from the site of the ischemic lesion. Employing Affymetrix GeneChip arrays, the gene expression profiling of reactive astrocytes by Zamanian and colleagues [53] provided transcriptome databases for two subtypes of reactive astrocytes that will be highly useful in generating new and testable hypotheses of their function, as well as for providing new markers to detect different types of reactive astrocytes.

### Co-expression of PDGFαR and GFAP in EGFP^+^ Cells

Our finding that at P10 almost all EGFP^+^ cells express *Cspg4* and *Pdgfra* and that EGFP^+^ cells co-express *Gfap* and *Cspg4/Pdgfra* was confirmed by immunohistochemistry. (Figure 4, Figure S3); [35]. Similarly, Cahoy and co-authors [25] also detected the high expression of *Cspg4* in immature astrocytes (P7, P17), which declined with astrocyte maturation; however, they never detected *Pdgfra* expression in astrocytes, but rather *Pdgfrb*, which is known to be expressed in pericytes [80]. Although GFAP is a traditional marker of astrocytes and we collected cells where the expression of EGFP is driven by a GFAP promoter, its mRNA level was very low, not at all comparable with the expression of *Cspg4* and *Pdgfra*. The GFAP protein must therefore be stable with slow turnover in the cortex. Nevertheless, *Gfap* and *Gfapδ* increased with astrocyte maturation similarly to the highly expressed *Glul, Eaat1* and *Aqp4*. Interestingly, in subpopulations A1, B2 and B3, *Cspg4* and *Pdgfra* are co-expressed on the mRNA level in 95% of cells, but the correlation between their expressions on the single cell level is not particularly strong (r = 0.44, p = 0.001 for A1, r = 0.23, p = 0.151 for B2, r = 0.27, p = 0.159 for B3). *Cspg4* and *Pdgfra* also show quite different correlations with other genes. This suggests *Cspg4* and *Pdgfra* are expressed independently of each other in individual cells, even in cells from the same subpopulation. The absence of correlation between highly expressed genes in individual cells of the same kind has been observed previously [33] and has been interpreted as the cells having independent transcription regulatory mechanisms, which leads to independent transcriptional bursts.

We do not think that the expression of *Cspg4* or *Pdgfra* in EGFP/GFAP cells isolated from the cortex of P10 animals was detected simply due to sensitivity in which low transcript levels are amplified. PDGFαR and NG2 are expressed in these cells also on the protein level as demonstrated by immunohistochemistry in Figure 4 for PDGFαR and in Figure S3 for NG2. These images show without any doubt that EGFP-positive cells (P10–20 mice) co-express GFAP and PDGFαR or NG2, the expression of which disappears at P50. More interestingly, in post-ischemic tissue the expression of both *Cspg4* and *Pdgfra* reappears; however, their expression on the protein level does not coincide with EGFP/GFAP-positive reactive astrocytes, which might suggest that newly generated reactive astrocytes lose both NG2 or PDGFαR when they reach the stage of fully developed reactive astroglia 14 days post ischemia, while those still expressing NG2 and/or PDGFαR might partially comprise cells in transition into reactive astrocytes or dedifferentiated astrocytes that display the gene expression profile of immature P10-P20 astrocytes (A1 subpopulation). This is also obvious from the division of the B3 subpopulation into two groups: B3A and B3B (Figure 6). Cells in the B3A group express *Cspg4* and *Pdgfra*, while the expression of *Eaat1, Glul* and *Aqp4* is significantly lower than in the B3B group. Therefore, NG2 or PDGFαR-positive cells may reflect astrocytes in a transitional stage similar to that described by Zhu and colleagues [81]. They showed that a subpopulation of GS^+^ astrocytes is the progeny of NG2 cells, thus confirming the previous finding that NG2 cells give rise to protoplasmic astrocytes. They also showed the co-expression of aldehyde dehydrogenase (*Aldh1L*1, another marker of astrocytes) immunoreactivity in their somata and faint PDGFαR immunoreactivity in their processes, indicative of transitional cells that are losing PDGFαR and acquiring Aldh1L1. Similar data showing the co-expression of GFAP and NG2 in ischemic tissue were described in our recent publication [12].

### The Basis of Astrocyte Heterogeneity

The heterogeneity of astrocytes even within an individual brain region, such as the cortex, might already arise during their development. Some astrocytes originate from cells in the ventricular zone via radial glia intermediates, while other astrocytes arise from immature cells that migrate in the perinatal period from the dorso-ventral subventricular zone into the brain [82]. Radial glia, which give rise to distinct subsets of forebrain neurons and later on to glial cells, must be highly heterogeneous themselves with respect to their progenitor function, which strongly depends on the expression of different sets of transcription factors such as Emx1 and Dach1. Progenitors expressing these transcription factors give rise, besides cortical and hippocampal CA1 neurons, to a subset of cortical and hippocampal astrocytes [83]–[85] and also to a subpopulation of astroglia in the dorsal roof of the subventricular zone [86]. How the developmental heterogeneity of astrocytes relates to their functional heterogeneity still remains unresolved. In addition, astrocytes might also display a spatial heterogeneity as they surround blood vessels and/or different synapses. Nonetheless, a diversity of astrocytic responses to pathological stimuli, such as oxygen-glucose deprivation or hypotonic stress, has been demonstrated in the cortex [5], [87]. As reported earlier [88], there also exists a subpopulation of astrocytes that do not express GFAP and that were not considered in this study.

### Conclusions

We have used single cell expression profiling and multivariate expression analysis to identify and characterize subpopulations of astrocytes present during postnatal development and after focal cerebral ischemia. We found that transcriptional activity decreases during development and that fully mature astrocytes express mainly *Eaat1, Glul, Aqp4* and *Kcnj10* and *Kcnj16*. After a brain injury the situation rapidly changes - the astrocytes become reactive and express *Eaat1, Glul, Aqp1, Aqp9, Snap25,* hyperpolarization-activated cation channels *Hcn1, Hcn2, Hcn3*, glutamate receptors *Grin2a, Gria2, Gria3, Grm5* and potassium channels *Kcna3* and *Vim*.

## Supporting Information

Figure S1 **Validation of pre-amplification.** Three separate RNA samples were isolated from the cortex of three different mice, transcribed and preamplified. cDNA levels were measured with preamplification (P) and without preamplification (NP). The average difference (ΔCq) and the standard deviation of the difference between P and NP for each gene were calculated for each of the three RNA samples.
(TIF)

Figure S2 **Schemas of all correlations higher than 0.6 for subpopulation A1 (A), subpopulation A2 (B), subpopulation A3 (C), subpopulation B1 (D), subpopulation B2 (E), and subpopulation B3 (F).** Positive correlations are indicated by green lines, while red lines indicate negative correlations.
(TIF)

Figure S3 **Immunohistochemical analysis of EGFP^+^ cells.** The expression of GFAP and NG2 was analyzed in the cortex of 10 days-old EGFP/GFAP mice. Note that at P10 there are EGFP-positive cells co-expressing GFAP and NG2.
(TIF)

Figure S4 **Interactions between highly expressed “key” genes in subpopulation B3.** (**A**) All significant correlations (p<0.05) between the expressions of gene pairs are indicated. (**B**) Partial Spearman correlation coefficients were calculated to separate direct and indirect interactions between genes. The lines indicate a direct correlation that remains significant after the removal of indirect (via a third gene) correlations. (**C**) Correlation that remains significant after removing the effect of Hcn2. (**D**) Correlation that remains significant after removing the effect of Gria3.
(TIF)

Table S1 **PCR assay information and primer sequences.**
(DOCX)

Table S2 **Statistics describing the gene expression for each postnatal developmental subpopulation or post-ischemic subpopulations.**
(DOCX)

Table S3 **Correlation within development subpopulation A1.**
(XLSX)

Table S4 **Correlation within development subpopulation A2.**
(XLSX)

Table S5 **Correlation within development subpopulation A3.**
(XLSX)

Table S6 **Correlation within development subpopulation B1.**
(XLSX)

Table S7 **Correlation within development subpopulation B2.**
(XLSX)

Table S8 **Correlation within development subpopulation B3.**
(XLSX)

## References

[1] Reichenbach WH (2005) Astrocytes and ependymal cells. In: Kettenmann H, Ransom BR, editor. Neuroglia. 2 ed. New York: Oxford University Press. 19–36.

[2] Oberheim NA, Takano T, Han X, He W, Lin JH, et al (2009) Uniquely hominid features of adult human astrocytes. The Journal of neuroscience : the official journal of the Society for Neuroscience 29: 3276–3287. 19279265 10.1523/JNEUROSCI.4707-08.2009 PMC2819812

[3] Matyash V, Kettenmann H (2010) Heterogeneity in astrocyte morphology and physiology. Brain research reviews 63: 2–10. 20005253 10.1016/j.brainresrev.2009.12.001

[4] Seifert G, Huttmann K, Binder DK, Hartmann C, Wyczynski A, et al (2009) Analysis of astroglial K+ channel expression in the developing hippocampus reveals a predominant role of the Kir4.1 subunit. The Journal of neuroscience : the official journal of the Society for Neuroscience 29: 7474–7488. 19515915 10.1523/JNEUROSCI.3790-08.2009 PMC6665420

[5] Benesova J, Rusnakova V, Honsa P, Pivonkova H, Dzamba D, et al (2012) Distinct expression/function of potassium and chloride channels contributes to the diverse volume regulation in cortical astrocytes of GFAP/EGFP mice. PLoS One 7: e29725. 22253765 10.1371/journal.pone.0029725 PMC3256164

[6] Ge WP, Miyawaki A, Gage FH, Jan YN, Jan LY (2012) Local generation of glia is a major astrocyte source in postnatal cortex. Nature 484: 376–380. 22456708 10.1038/nature10959 PMC3777276

[7] Freeman MR (2010) Specification and morphogenesis of astrocytes. Science 330: 774–778. 21051628 10.1126/science.1190928 PMC5201129

[8] Zhang Y, Barres BA (2010) Astrocyte heterogeneity: an underappreciated topic in neurobiology. Current opinion in neurobiology 20: 588–594. 20655735 10.1016/j.conb.2010.06.005

[9] Trotter J, Karram K, Nishiyama A (2010) NG2 cells: Properties, progeny and origin. Brain research reviews 63: 72–82. 20043946 10.1016/j.brainresrev.2009.12.006 PMC2862831

[10] Stallcup WB, Beasley L (1987) Bipotential glial precursor cells of the optic nerve express the NG2 proteoglycan. The Journal of neuroscience : the official journal of the Society for Neuroscience 7: 2737–2744. 3305800 10.1523/JNEUROSCI.07-09-02737.1987 PMC6569154

[11] Alonso G (2005) NG2 proteoglycan-expressing cells of the adult rat brain: possible involvement in the formation of glial scar astrocytes following stab wound. Glia 49: 318–338. 15494983 10.1002/glia.20121

[12] Honsa P, Pivonkova H, Dzamba D, Filipova M, Anderova M (2012) Polydendrocytes display large lineage plasticity following focal cerebral ischemia. PLoS One 7: e36816. 22590616 10.1371/journal.pone.0036816 PMC3349640

[13] Robel S, Berninger B, Gotz M (2011) The stem cell potential of glia: lessons from reactive gliosis. Nature reviews Neuroscience 12: 88–104. 21248788 10.1038/nrn2978

[14] Zelenina M (2010) Regulation of brain aquaporins. Neurochemistry international 57: 468–488. 20380861 10.1016/j.neuint.2010.03.022

[15] Hwang IK, Yoo KY, Li H, Lee BH, Suh HW, et al (2007) Aquaporin 9 changes in pyramidal cells before and is expressed in astrocytes after delayed neuronal death in the ischemic hippocampal CA1 region of the gerbil. Journal of neuroscience research 85: 2470–2479. 17526024 10.1002/jnr.21381

[16] McKeon RJ, Jurynec MJ, Buck CR (1999) The chondroitin sulfate proteoglycans neurocan and phosphacan are expressed by reactive astrocytes in the chronic CNS glial scar. The Journal of neuroscience : the official journal of the Society for Neuroscience 19: 10778–10788. 10594061 10.1523/JNEUROSCI.19-24-10778.1999 PMC6784959

[17] Komitova M, Perfilieva E, Mattsson B, Eriksson PS, Johansson BB (2002) Effects of cortical ischemia and postischemic environmental enrichment on hippocampal cell genesis and differentiation in the adult rat. Journal of cerebral blood flow and metabolism : official journal of the International Society of Cerebral Blood Flow and Metabolism 22: 852–860. 10.1097/00004647-200207000-00010 12142570

[18] Ferraguti F, Corti C, Valerio E, Mion S, Xuereb J (2001) Activated astrocytes in areas of kainate-induced neuronal injury upregulate the expression of the metabotropic glutamate receptors 2/3 and 5. Experimental brain research Experimentelle Hirnforschung Experimentation cerebrale 137: 1–11. 11310162 10.1007/s002210000633

[19] Ulas J, Satou T, Ivins KJ, Kesslak JP, Cotman CW, et al (2000) Expression of metabotropic glutamate receptor 5 is increased in astrocytes after kainate-induced epileptic seizures. Glia 30: 352–361. 10797615

[20] Jacque CM, Vinner C, Kujas M, Raoul M, Racadot J, et al (1978) Determination of glial fibrillary acidic protein (GFAP) in human brain tumors. Journal of the neurological sciences 35: 147–155. 624958 10.1016/0022-510x(78)90107-7

[21] Sofroniew MV, Vinters HV (2010) Astrocytes: biology and pathology. Acta neuropathologica 119: 7–35. 20012068 10.1007/s00401-009-0619-8 PMC2799634

[22] Walz W (2000) Controversy surrounding the existence of discrete functional classes of astrocytes in adult gray matter. Glia 31: 95–103. 10878596 10.1002/1098-1136(200008)31:2<95::aid-glia10>3.0.co;2-6

[23] Regan MR, Huang YH, Kim YS, Dykes-Hoberg MI, Jin L, et al (2007) Variations in promoter activity reveal a differential expression and physiology of glutamate transporters by glia in the developing and mature CNS. The Journal of neuroscience : the official journal of the Society for Neuroscience 27: 6607–6619. 17581948 10.1523/JNEUROSCI.0790-07.2007 PMC6672708

[24] Mearow KM, Mill JF, Vitkovic L (1989) The ontogeny and localization of glutamine synthetase gene expression in rat brain. Brain research Molecular brain research 6: 223–232. 2574402 10.1016/0169-328x(89)90068-5

[25] Cahoy JD, Emery B, Kaushal A, Foo LC, Zamanian JL, et al (2008) A transcriptome database for astrocytes, neurons, and oligodendrocytes: a new resource for understanding brain development and function. The Journal of neuroscience : the official journal of the Society for Neuroscience 28: 264–278. 18171944 10.1523/JNEUROSCI.4178-07.2008 PMC6671143

[26] Kimelberg HK (2004) The problem of astrocyte identity. Neurochemistry international 45: 191–202. 15145537 10.1016/j.neuint.2003.08.015

[27] Klein CA, Zohlnhofer D, Petat-Dutter K, Wendler N (2005) Gene expression analysis of a single or few cells. Current protocols in human genetics/editorial board, Jonathan L Haines [et al] Chapter 11: Unit 11 18. 10.1002/0471142905.hg1108s44 18428372

[28] Ståhlberg A, Andersson D, Aurelius J, Faiz M, Pekna M, et al (2011) Defining cell populations with single-cell gene expression profiling: correlations and identification of astrocyte subpopulations. Nucleic acids research 39: e24. 21112872 10.1093/nar/gkq1182 PMC3045576

[29] Raj A, Peskin CS, Tranchina D, Vargas DY, Tyagi S (2006) Stochastic mRNA synthesis in mammalian cells. PLoS biology 4: e309. 17048983 10.1371/journal.pbio.0040309 PMC1563489

[30] Raj A, van Oudenaarden A (2009) Single-molecule approaches to stochastic gene expression. Annual review of biophysics 38: 255–270. 10.1146/annurev.biophys.37.032807.125928 PMC3126657 19416069

[31] Bengtsson M, Hemberg M, Rorsman P, Stahlberg A (2008) Quantification of mRNA in single cells and modelling of RT-qPCR induced noise. BMC molecular biology 9: 63. 18631407 10.1186/1471-2199-9-63 PMC2483285

[32] Ståhlberg A, Rusnakova V, Forootan A, Anderova M, Kubista M (2013) RT-qPCR work-flow for single-cell data analysis. Methods 59: 80–88. 23021995 10.1016/j.ymeth.2012.09.007

[33] Ståhlberg A, Bengtsson M (2010) Single-cell gene expression profiling using reverse transcription quantitative real-time PCR. Methods 50: 282–288. 20064613 10.1016/j.ymeth.2010.01.002

[34] Nolte C, Matyash M, Pivneva T, Schipke CG, Ohlemeyer C, et al (2001) GFAP promoter-controlled EGFP-expressing transgenic mice: a tool to visualize astrocytes and astrogliosis in living brain tissue. Glia 33: 72–86. 11169793

[35] Benesova J, Hock M, Butenko O, Prajerova I, Anderova M, et al (2009) Quantification of astrocyte volume changes during ischemia in situ reveals two populations of astrocytes in the cortex of GFAP/EGFP mice. Journal of neuroscience research 87: 96–111. 18752295 10.1002/jnr.21828

[36] Wahl-Schott C, Biel M (2009) HCN channels: structure, cellular regulation and physiological function. Cell Mol Life Sci 66: 470–494. 18953682 10.1007/s00018-008-8525-0 PMC11131499

[37] Ståhlberg A, Rusnakova V, Kubista M (2013) The added value of single-cell gene expression profiling. Brief Funct Genomics. 10.1093/bfgp/elt001 23393397

[38] Sindelka R, Ferjentsik Z, Jonak J (2006) Developmental expression profiles of Xenopus laevis reference genes. Dev Dyn 235: 754–758. 16397894 10.1002/dvdy.20665

[39] da Cunha A, Aloyo VJ, Vitkovic L (1991) Developmental regulation of GAP-43, glutamine synthetase and beta-actin mRNA in rat cortical astrocytes. Brain research Developmental brain research 64: 212–215. 1686218 10.1016/0165-3806(91)90228-b

[40] Stanimirovic DB, Ball R, Small DL, Muruganandam A (1999) Developmental regulation of glutamate transporters and glutamine synthetase activity in astrocyte cultures differentiated in vitro. International journal of developmental neuroscience : the official journal of the International Society for Developmental Neuroscience 17: 173–184. 10452361 10.1016/s0736-5748(99)00028-3

[41] Palygin O, Lalo U, Pankratov Y (2011) Distinct pharmacological and functional properties of NMDA receptors in mouse cortical astrocytes. Br J Pharmacol 163: 1755–1766. 21449975 10.1111/j.1476-5381.2011.01374.x PMC3166701

[42] Ziak D, Chvatal A, Sykova E (1998) Glutamate-, kainate- and NMDA-evoked membrane currents in identified glial cells in rat spinal cord slice. Physiological research/Academia Scientiarum Bohemoslovaca 47: 365–375. 10052606

[43] Matthias K, Kirchhoff F, Seifert G, Huttmann K, Matyash M, et al (2003) Segregated expression of AMPA-type glutamate receptors and glutamate transporters defines distinct astrocyte populations in the mouse hippocampus. The Journal of neuroscience : the official journal of the Society for Neuroscience 23: 1750–1758. 12629179 10.1523/JNEUROSCI.23-05-01750.2003 PMC6741945

[44] Butt AM, Hamilton N, Hubbard P, Pugh M, Ibrahim M (2005) Synantocytes: the fifth element. Journal of anatomy 207: 695–706. 16367797 10.1111/j.1469-7580.2005.00458.x PMC1571581

[45] Zhou M (2006) Schools GP, Kimelberg HK (2006) Development of GLAST(+) astrocytes and NG2(+) glia in rat hippocampus CA1: mature astrocytes are electrophysiologically passive. Journal of neurophysiology 95: 134–143. 16093329 10.1152/jn.00570.2005

[46] Nagelhus EA, Horio Y, Inanobe A, Fujita A, Haug FM, et al (1999) Immunogold evidence suggests that coupling of K+ siphoning and water transport in rat retinal Muller cells is mediated by a coenrichment of Kir4.1 and AQP4 in specific membrane domains. Glia 26: 47–54. 10088671 10.1002/(sici)1098-1136(199903)26:1<47::aid-glia5>3.0.co;2-5

[47] Connors NC, Adams ME, Froehner SC, Kofuji P (2004) The potassium channel Kir4.1 associates with the dystrophin-glycoprotein complex via alpha-syntrophin in glia. The Journal of biological chemistry 279: 28387–28392. 15102837 10.1074/jbc.M402604200

[48] Liu H, Yang M, Qiu GP, Zhuo F, Yu WH, et al (2012) Aquaporin 9 in rat brain after severe traumatic brain injury. Arquivos de neuro-psiquiatria 70: 214–220. 22392116 10.1590/s0004-282x2012000300012

[49] Sun W, McConnell E, Pare JF, Xu Q, Chen M, et al (2013) Glutamate-dependent neuroglial calcium signaling differs between young and adult brain. Science 339: 197–200. 23307741 10.1126/science.1226740 PMC3569008

[50] Lyon L, Kew JN, Corti C, Harrison PJ, Burnet PW (2008) Altered hippocampal expression of glutamate receptors and transporters in GRM2 and GRM3 knockout mice. Synapse 62: 842–850. 18720515 10.1002/syn.20553 PMC2673354

[51] Masood K, Besnard F, Su Y, Brenner M (1993) Analysis of a segment of the human glial fibrillary acidic protein gene that directs astrocyte-specific transcription. J Neurochem 61: 160–166. 8515262 10.1111/j.1471-4159.1993.tb03551.x

[52] Pekny M, Nilsson M (2005) Astrocyte activation and reactive gliosis. Glia 50: 427–434. 15846805 10.1002/glia.20207

[53] Zamanian JL, Xu L, Foo LC, Nouri N, Zhou L, et al (2012) Genomic analysis of reactive astrogliosis. The Journal of neuroscience : the official journal of the Society for Neuroscience 32: 6391–6410. 22553043 10.1523/JNEUROSCI.6221-11.2012 PMC3480225

[54] Hoshi A, Yamamoto T, Shimizu K, Ugawa Y, Nishizawa M, et al (2012) Characteristics of aquaporin expression surrounding senile plaques and cerebral amyloid angiopathy in Alzheimer disease. J Neuropathol Exp Neurol 71: 750–759. 22805778 10.1097/NEN.0b013e3182632566

[55] McCoy E, Sontheimer H (2010) MAPK induces AQP1 expression in astrocytes following injury. Glia 58: 209–217. 19610096 10.1002/glia.20916 PMC2992951

[56] Rodriguez A, Perez-Gracia E, Espinosa JC, Pumarola M, Torres JM, et al (2006) Increased expression of water channel aquaporin 1 and aquaporin 4 in Creutzfeldt-Jakob disease and in bovine spongiform encephalopathy-infected bovine-PrP transgenic mice. Acta Neuropathol 112: 573–585. 16871401 10.1007/s00401-006-0117-1

[57] Badaut J, Petit JM, Brunet JF, Magistretti PJ, Charriaut-Marlangue C, et al (2004) Distribution of Aquaporin 9 in the adult rat brain: preferential expression in catecholaminergic neurons and in glial cells. Neuroscience 128: 27–38. 15450351 10.1016/j.neuroscience.2004.05.042

[58] Badaut J, Regli L (2004) Distribution and possible roles of aquaporin 9 in the brain. Neuroscience 129: 971–981. 15561412 10.1016/j.neuroscience.2004.06.035

[59] Hara-Chikuma M, Verkman AS (2006) Aquaporin-1 facilitates epithelial cell migration in kidney proximal tubule. J Am Soc Nephrol 17: 39–45. 16319186 10.1681/ASN.2005080846

[60] McCoy E, Sontheimer H (2007) Expression and function of water channels (aquaporins) in migrating malignant astrocytes. Glia 55: 1034–1043. 17549682 10.1002/glia.20524 PMC2561225

[61] Papadopoulos MC, Verkman AS (2008) Potential utility of aquaporin modulators for therapy of brain disorders. Prog Brain Res 170: 589–601. 18655912 10.1016/S0079-6123(08)00446-9 PMC3601944

[62] Bezzi P, Carmignoto G, Pasti L, Vesce S, Rossi D, et al (1998) Prostaglandins stimulate calcium-dependent glutamate release in astrocytes. Nature 391: 281–285. 9440691 10.1038/34651

[63] Paquet M, Ribeiro FM, Guadagno J, Esseltine JL, Ferguson SS, et al (2013) Role of metabotropic glutamate receptor 5 signaling and homer in oxygen glucose deprivation-mediated astrocyte apoptosis. Mol Brain 6: 9. 23406666 10.1186/1756-6606-6-9 PMC3598502

[64] Durand D, Carniglia L, Caruso C, Lasaga M (2011) Reduced cAMP, Akt activation and p65-c-Rel dimerization: mechanisms involved in the protective effects of mGluR3 agonists in cultured astrocytes. PLoS One 6: e22235. 21779400 10.1371/journal.pone.0022235 PMC3136520

[65] Aguado F, Espinosa-Parrilla JF, Carmona MA, Soriano E (2002) Neuronal activity regulates correlated network properties of spontaneous calcium transients in astrocytes in situ. J Neurosci 22: 9430–9444. 12417668 10.1523/JNEUROSCI.22-21-09430.2002 PMC6758057

[66] Anderova M, Vorisek I, Pivonkova H, Benesova J, Vargova L, et al (2011) Cell death/proliferation and alterations in glial morphology contribute to changes in diffusivity in the rat hippocampus after hypoxia-ischemia. J Cereb Blood Flow Metab 31: 894–907. 20877389 10.1038/jcbfm.2010.168 PMC3063622

[67] Sik A, Smith RL, Freund TF (2000) Distribution of chloride channel-2-immunoreactive neuronal and astrocytic processes in the hippocampus. Neuroscience 101: 51–65. 11068136 10.1016/s0306-4522(00)00360-2

[68] Ransom CB, O'Neal JT, Sontheimer H (2001) Volume-activated chloride currents contribute to the resting conductance and invasive migration of human glioma cells. J Neurosci 21: 7674–7683. 11567057 10.1523/JNEUROSCI.21-19-07674.2001 PMC6762888

[69] Butenko O, Dzamba D, Benesova J, Honsa P, Benfenati V, et al (2012) The increased activity of TRPV4 channel in the astrocytes of the adult rat hippocampus after cerebral hypoxia/ischemia. PLoS One 7: e39959. 22761937 10.1371/journal.pone.0039959 PMC3384594

[70] Benfenati V, Amiry-Moghaddam M, Caprini M, Mylonakou MN, Rapisarda C, et al (2007) Expression and functional characterization of transient receptor potential vanilloid-related channel 4 (TRPV4) in rat cortical astrocytes. Neuroscience 148: 876–892. 17719182 10.1016/j.neuroscience.2007.06.039

[71] Pamenter ME, Perkins GA, McGinness AK, Gu XQ, Ellisman MH, et al (2012) Autophagy and apoptosis are differentially induced in neurons and astrocytes treated with an in vitro mimic of the ischemic penumbra. PLoS One 7: e51469. 23251543 10.1371/journal.pone.0051469 PMC3520810

[72] Neitz A, Mergia E, Eysel UT, Koesling D, Mittmann T (2011) Presynaptic nitric oxide/cGMP facilitates glutamate release via hyperpolarization-activated cyclic nucleotide-gated channels in the hippocampus. Eur J Neurosci 33: 1611–1621. 21410795 10.1111/j.1460-9568.2011.07654.x

[73] Zhao JW, Raha-Chowdhury R, Fawcett JW, Watts C (2009) Astrocytes and oligodendrocytes can be generated from NG2+ progenitors after acute brain injury: intracellular localization of oligodendrocyte transcription factor 2 is associated with their fate choice. The European journal of neuroscience 29: 1853–1869. 19473238 10.1111/j.1460-9568.2009.06736.x

[74] Komitova M, Serwanski DR, Lu QR, Nishiyama A (2011) NG2 cells are not a major source of reactive astrocytes after neocortical stab wound injury. Glia 59: 800–809. 21351161 10.1002/glia.21152 PMC3560299

[75] Patanow CM, Day JR, Billingsley ML (1997) Alterations in hippocampal expression of SNAP-25, GAP-43, stannin and glial fibrillary acidic protein following mechanical and trimethyltin-induced injury in the rat. Neuroscience 76: 187–202. 8971771 10.1016/s0306-4522(96)00335-1

[76] Hepp R, Perraut M, Chasserot-Golaz S, Galli T, Aunis D, et al (1999) Cultured glial cells express the SNAP-25 analogue SNAP-23. Glia 27: 181–187. 10417817 10.1002/(sici)1098-1136(199908)27:2<181::aid-glia8>3.0.co;2-9

[77] Illarionova NB, Gunnarson E, Li Y, Brismar H, Bondar A, et al (2010) Functional and molecular interactions between aquaporins and Na,K-ATPase. Neuroscience 168: 915–925. 19962432 10.1016/j.neuroscience.2009.11.062

[78] Anderova M, Antonova T, Petrik D, Neprasova H, Chvatal A, et al (2004) Voltage-dependent potassium currents in hypertrophied rat astrocytes after a cortical stab wound. Glia 48: 311–326. 15390116 10.1002/glia.20076

[79] Bordey A, Lyons SA, Hablitz JJ, Sontheimer H (2001) Electrophysiological characteristics of reactive astrocytes in experimental cortical dysplasia. Journal of neurophysiology 85: 1719–1731. 11287494 10.1152/jn.2001.85.4.1719

[80] Arimura K, Ago T, Kamouchi M, Nakamura K, Ishitsuka K, et al (2012) PDGF receptor beta signaling in pericytes following ischemic brain injury. Curr Neurovasc Res 9: 1–9. 22272762 10.2174/156720212799297100

[81] Zhu X, Zuo H, Maher BJ, Serwanski DR, LoTurco JJ, et al (2012) Olig2-dependent developmental fate switch of NG2 cells. Development 139: 2299–2307. 22627280 10.1242/dev.078873 PMC3367441

[82] Marshall CA, Suzuki SO, Goldman JE (2003) Gliogenic and neurogenic progenitors of the subventricular zone: who are they, where did they come from, and where are they going? Glia 43: 52–61. 12761867 10.1002/glia.10213

[83] Gorski JA, Talley T, Qiu M, Puelles L, Rubenstein JL, et al (2002) Cortical excitatory neurons and glia, but not GABAergic neurons, are produced in the Emx1-expressing lineage. J Neurosci 22: 6309–6314. 12151506 10.1523/JNEUROSCI.22-15-06309.2002 PMC6758181

[84] Machon O, van den Bout CJ, Backman M, Rosok O, Caubit X, et al (2002) Forebrain-specific promoter/enhancer D6 derived from the mouse Dach1 gene controls expression in neural stem cells. Neuroscience 112: 951–966. 12088753 10.1016/s0306-4522(02)00053-2

[85] Prajerova I, Honsa P, Chvatal A, Anderova M (2010) Neural stem/progenitor cells derived from the embryonic dorsal telencephalon of D6/GFP mice differentiate primarily into neurons after transplantation into a cortical lesion. Cell Mol Neurobiol 30: 199–218. 19707869 10.1007/s10571-009-9443-x PMC11498807

[86] Honsa P, Pivonkova H, Anderova M (2013) Focal cerebral ischemia induces the neurogenic potential of mouse Dach1-expressing cells in the dorsal part of the lateral ventricles. Neuroscience. 10.1016/j.neuroscience.2013.02.048 23458709

[87] Benesova J, Hock M, Butenko O, Prajerova I, Anderova M, et al (2009) Quantification of astrocyte volume changes during ischemia in situ reveals two populations of astrocytes in the cortex of GFAP/EGFP mice. J Neurosci Res 87: 96–111. 18752295 10.1002/jnr.21828

[88] Walz W, Lang MK (1998) Immunocytochemical evidence for a distinct GFAP-negative subpopulation of astrocytes in the adult rat hippocampus. Neurosci Lett. 257(3): 127–30. 10.1016/s0304-3940(98)00813-1 9870336

