# Supplementary figures and images for: Heterogeneity of Astrocytes: From Development to Injury – Single Cell Gene Expression
Source: PLoS One. 2013 Aug 5;8(8):e69734. doi: 10.1371/journal.pone.0069734 (PMC3734191; doi:10.1371/journal.pone.0069734)

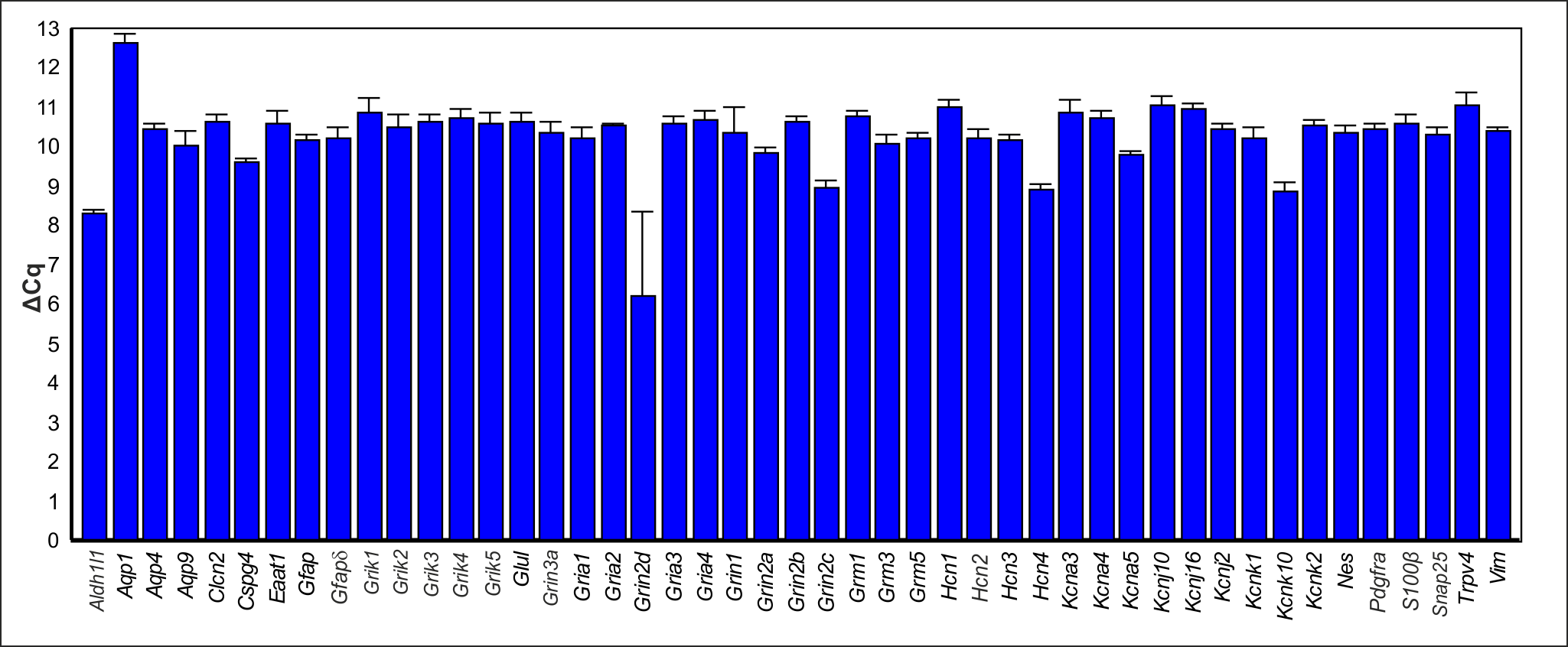

Supplement: Figure S1 — Validation of pre-amplification. Three separate RNA samples were isolated from the cortex of three different mice, transcribed and preamplified. cDNA levels were measured with preamplification (P) and without preamplification (NP). The average difference (ΔCq) and the standard deviation of the difference between P and NP for each gene were calculated for each of the three RNA samples. (TIF) [file pone.0069734.s001.tif]

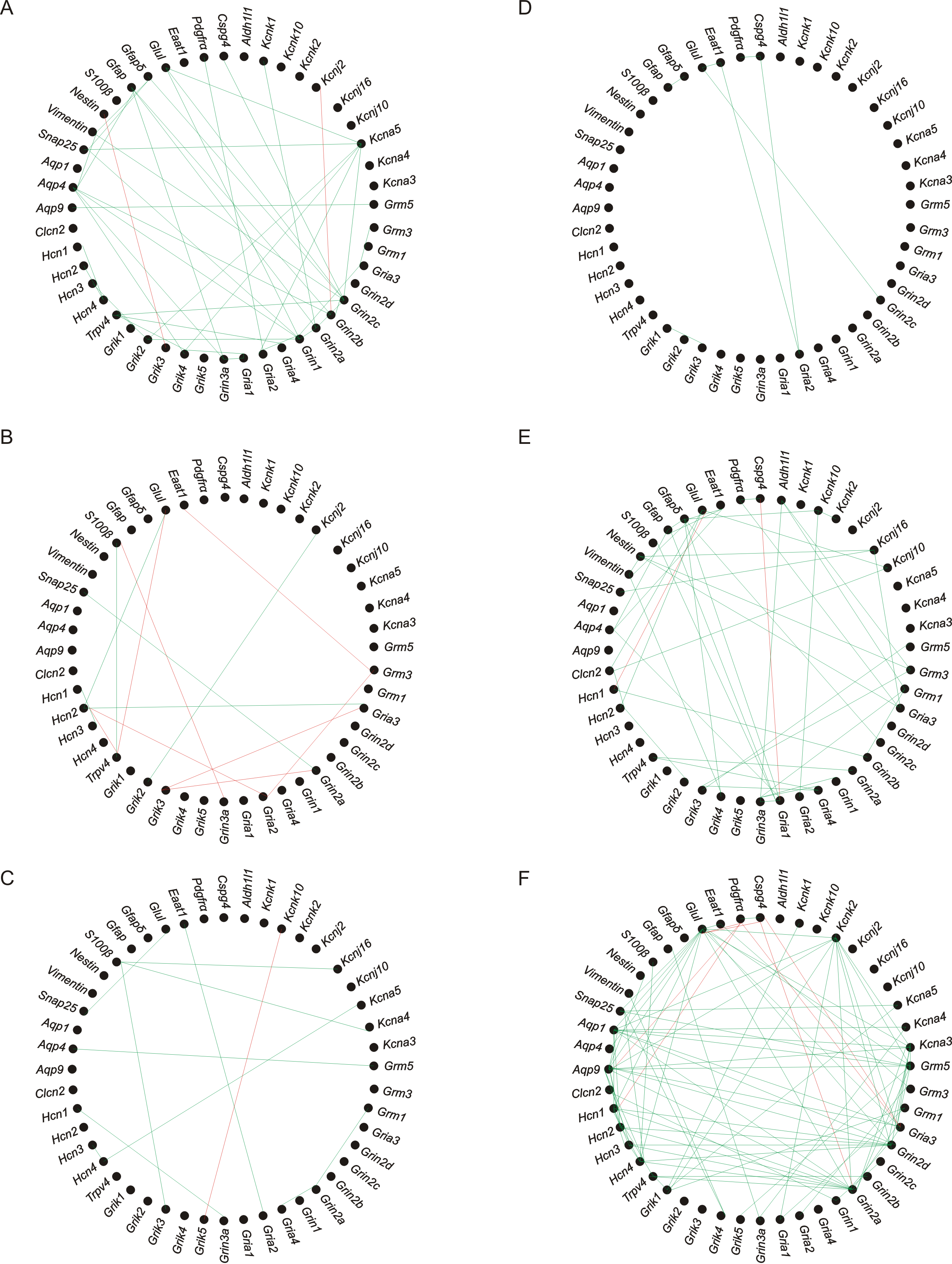

Supplement: Figure S2 — Schemas of all correlations higher than 0.6 for subpopulation A1 (A), subpopulation A2 (B), subpopulation A3 (C), subpopulation B1 (D), subpopulation B2 (E), and subpopulation B3 (F). Positive correlations are indicated by green lines, while red lines indicate negative correlations. (TIF) [file pone.0069734.s002.tif]

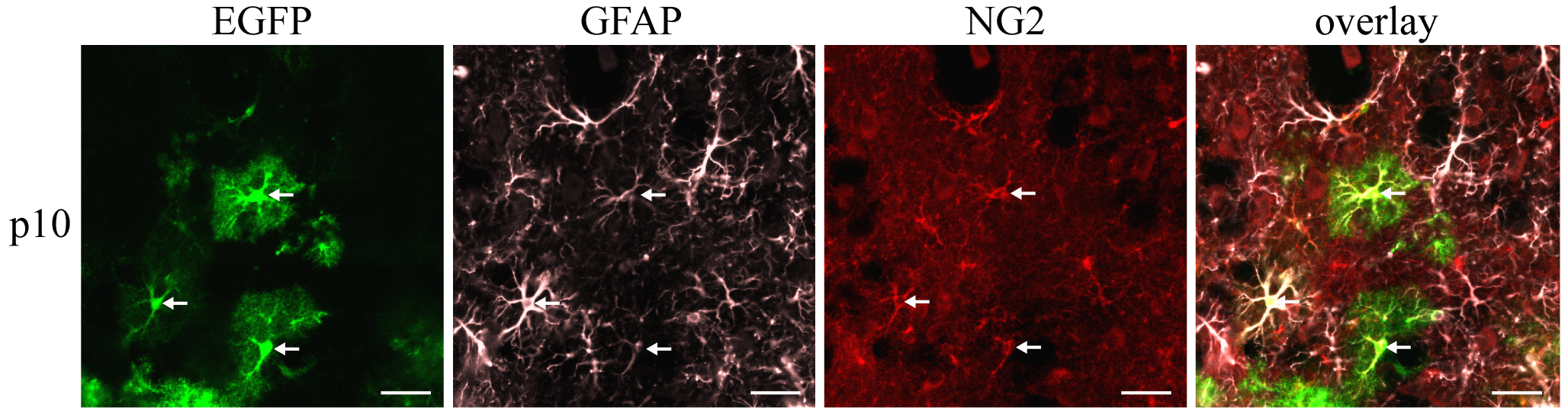

Supplement: Figure S3 — Immunohistochemical analysis of EGFP+ cells. The expression of GFAP and NG2 was analyzed in the cortex of 10 days-old EGFP/GFAP mice. Note that at P10 there are EGFP-positive cells co-expressing GFAP and NG2. (TIF) [file pone.0069734.s003.tif]

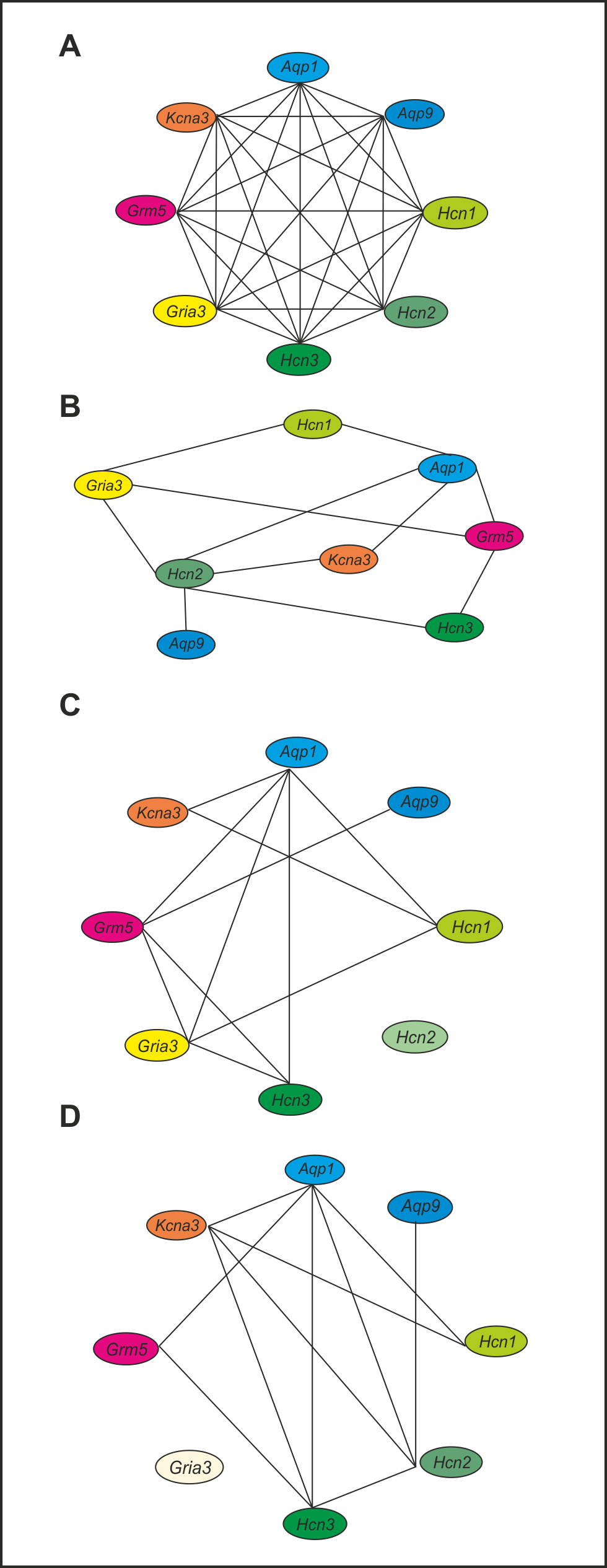

Supplement: Figure S4 — Interactions between highly expressed “key” genes in subpopulation B3. (A) All significant correlations (p<0.05) between the expressions of gene pairs are indicated. (B) Partial Spearman correlation coefficients were calculated to separate direct and indirect interactions between genes. The lines indicate a direct correlation that remains significant after the removal of indirect (via a third gene) correlations. (C) Correlation that remains significant after removing the effect of Hcn2. (D) Correlation that remains significant after removing the effect of Gria3. (TIF) [file pone.0069734.s004.tif]
