# Supplementary material for: Heterogeneity of Astrocytes: From Development to Injury – Single Cell Gene Expression
Source: PLoS One. 2013 Aug 5;8(8):e69734. doi: 10.1371/journal.pone.0069734 (PMC3734191; doi:10.1371/journal.pone.0069734)
Supplement: Table S1 — PCR assay information and primer sequences. (DOCX) [file pone.0069734.s005.docx]

**Table S1: PCR assay information and primer sequences.**

| **Gene/Protein** | **Efficiency** | **PUBMED ID** | **Forward primer** | **Reverse primer** |
| --- | --- | --- | --- | --- |
| *Aqp1*/AQP1 | 97 % | NM_007472.2 | CTGGCTGCGGTATCAACC | GGATGAAGTCATAGATGAGCACTG |
| *Aqp4*/AQP4 | 100 % | NM_009700.2 | CGGCATCCTCTACCTGGTCACA | GCCAGCGGTGAGGTTTCCAT |
| *Aqp9/*AQP9 | 100 % | NM_022026.2 | GAAGGATGGAGTGGTTCAAGTTC | TGGCACGGATACAAATGGTTT |
| *Clcn2*/ClC2 | 96 % | NM_009900.2 | TGCCAATGTCTTCCTTACTCTG | ATTCGGTAGGTGCTGCTATC |
| *Cspg4*/NG2 | 103 % | NM_139001.2 | TGATGGAAGTGAGACACAGACAGA | GGAAGGATGGTGATCGTGAAGG |
| *Eaat1*/GLAST | 98 % | NM_148938.3 | ATCGTCCTGCCTCTCCTCTAC | GTCCACACCATTGTTCTCTTCCA |
| *Gfap*/GFAP | 92 % | NM_001131020.1 | GAACAACCTGGCTGCGTATAG | GCGATTCAACCTTTCTCTCCAA |
| *Gfapδ*/GFAPδ | 96 % | NM_001131019.1 | ATGTGTCTCAGTTGTGAAGGTCTA | TGGAAGGATGGTTGTGGATTCT |
| *Grik1*/GluK1 | 94 % | NM_146072.4 | CACGAGACGGCTGCTGAA | ACCACTGTACCTGTAGAGTTCCA |
| *Grik2*/GluK2 | 92 % | NM_001111268.1 | ACAATCAACAGGAACAGGACTCT | TGCTGATGAACTGTGTGAAGGA |
| *Grik3*/GluK3 | 96 % | NM_001081097.2 | GCTCAGAGGTGGTGGAGAATA | GCCGTGTAGGAGGAGATGAT |
| *Grik4*/GluK4 | 90 % | NM_175481.5 | CGCATGGTAGAATTGGAAGGT | AAGAGACTGTCAGAGATGTTGGA |
| *Grik5*/GluK5 | 90 % | NM_008168.2 | CCACCTTGTCCTCCGTAA | CTCCACGATACCATCCAGAT |
| *Glul*/GS | 97 % | NM_008131.3 | CGCAAAGACCCCAACAAG | ATTCCTGCTCCATTCCAAAC |
| *Grin3a*/GluN3A | 91 % | NM_001033351.1 | GACAAAGCCCTTCTGGATTATGA | ATGTTAGAGGTCAACGGAGAGT |
| *Gria1*/GluA1 | 103 % | NM_001113325.1 | ACTCAAGCGTCCAGAATAGAAC | AATCTCAAGTCGGTAGGAATAGC |
| *Gria2*/GluA2 | 100 % | NM_001039195.1 | CAGATTGTAGACTACGACGACTC | TCATCACTTGGACAGCATCATAA |
| *Gria3*/GluA3 | 100 % | NM_016886.3 | GGTCATTCTCACGGAGGATTC | GGTGTTCTGGTTGGTGTTGTA |
| *Gria4*/GluA4 | 98 % | NM_001113180.1 | CCAGTAGAGGACAACGCAATT | TGACAGAGTGAAGGTTACAGGAA |
| *Grin1*/GluN1 | 96 % | NM_001177656.1 | AGATAGTGACAATCCACCAAGAAC | ACCATTGACTGTGAACTCCTC |
| *Grin2a*/GluN2A | 97 % | NM_008170.2 | GACCAGATGCTTCAGGAGACAG | CTTGAGGCTTATGCTACGAGAGG |
| *Grin2b*/GluN2B | 97 % | NM_008171.3 | GGTGTTTAACAACTCCGTACCT | GAAACCTGGTCCACATACTCCTC |
| *Grin2c/*GluN2C | 96 % | NM_010350.2 | CGTGTGGTTAGTACCTAATCTG | TTCTGGCGTAGGCTAAGG |
| *Grin2d/*GluN2D | 79 % | NM_008172.2 | AACCGAGACTACTCCTTCAATGA | GCCATAGCGGGACCATAGA |
| *Grm3*/mGluR3 | 96 % | NM_181850.2 | CGACCACATATTCTCAGTCCTCT | AGCACTTCGTCTAACAGCCTATA |
| *Grm5*/mGluR5 | 91% | NM_001081414.2 | CAGCTTAGATCGCAGCCACT | CAAGAATTTGGGTAAAATCACCA |
| *Hcn1*/HCN1 | 98 % | NM_010408.3 | CTCAGTCTCTTGCGGTTATTACG | TGGCGAGGTCATAGGTCAT |
| *Hcn2*/HCN2 | 96 % | NM_008226.2 | ATCGCATAGGCAAGAAGAACTC | CAATCTCCTGGATGATGGCATT |
| *Hcn3/*HCN3 | 94 % | NM_008227.1 | GATGTTTGATGAAGAGAGCATCC | CCCGGCAGGTGAAGTTAATA |
| *Hcn4*/HCN4 | 93 % | NM_001081192.1 | GCATGATGCTTCTGCTGTGT | GCTTCCCCCAGGAGTTATTC |
| *Kcna3*/Kv1.3 | 100 % | NM_008418.2 | GTAAGTCGGAGTATATGGTGAT | CAGTGAATATCTTCTTGATGTTGA |
| *Kcna4*/Kv1.4 | 105 % | NM_021275.4 | CTGAATGACACCTCGGCAC | AGCATCGAACCACAAACTCA |
| *Kcna5*/Kv1.5 | 104 % | NM_145983.2 | CGTGTCGGTCTTGGTCATTC | CGTAACAGCTCCCGTTCATC |
| *Kcnj10*/Kir4.1 | 94 % | NM_001039484.1 | AACTTGGGAGATTGAGATATGATATA | AAGTCTGAATACTTCCTTCTGTAC |
| *Kcnj16*/Kir5.1 | 93 % | NM_010604.3 | CCTGTGTCTCCTCTTGAAGG | TGTGCTTAGGTGATACAATACGG |
| *Kcnj2*/Kir2.1 | 99 % | NM_008425.4 | TCCCTCCCTTTCCCAAACAC | GAGGCTTGATTTTGAGACGC |
| *Kcnk1*/TWIK-1 | 97 % | NM_008430.2 | GGGAAATTGGAATTGGGACTTCA | TGCCGATGACAGAGTAGATGAT |
| *Kcnk10*/TREK-2 | 96 % | NM_029911.4 | CACTGTGGCTATCTCCTTAACC | GGCTGAGGCGGTGTAATC |
| *Kcnk2*/TREK-1 | 90 % | NM_010607.2 | ATTCGTATCATCTCCACCATCATC | CACACCACAGGCTTGTAGAA |
| *Grm1*/mGluR1 | 89 % | NM_001114333.2 | CGAGTGGAGTGACATAGAATC | TACCAGCCAGAATGATATAGCA |
| *Nes*/Nestin | 95 % | NM_016701.3 | AGCAACTGGCACACCTCAA | GGTATTAGGCAAGGGGGAAG |
| *Pdgfra*/PDGFαR | 97 % | NM_001083316.1 | AAGAGACCCTCCTTCTACCAC | TATCAGAGTCCACCCGCAT |
| *S100b/*S100β | 94 % | NM_009115.3 | TCTAACTCAGGACCGAGAATCA | GGAGCAAGGAAGATACAACTAACT |
| *Snap25/*SNAP25 | 93 % | NM_011428.3 | TCATCTGGTGGCTCTAATTCCTAA | AACAGCACATTGAGCATTCCTAA |
| *Vim*/Vimentin | 94 % | NM_011701.4 | TGCCAACCTTTTCTTCCCTG | TCTCTGGTCTCAACCGTCTT |
