# Supplementary material for: Heterogeneity of Astrocytes: From Development to Injury – Single Cell Gene Expression
Source: PLoS One. 2013 Aug 5;8(8):e69734. doi: 10.1371/journal.pone.0069734 (PMC3734191; doi:10.1371/journal.pone.0069734)
Supplement: Table S2 — Statistics describing the gene expression for each postnatal developmental subpopulation or post-ischemic subpopulations. (DOCX) [file pone.0069734.s006.docx]

**Table S2: Statistics describing the gene expression for each postnatal developmental subpopulation or post-ischemic subpopulations.**

|  | **Subpopulations of postnatal development** | | | | | | | | | | |  | **Subpopulations of all post-ischemic stages and P50** | | | | | | | | | | | | | |
| --- | --- | --- | --- | --- | --- | --- | --- | --- | --- | --- | --- | --- | --- | --- | --- | --- | --- | --- | --- | --- | --- | --- | --- | --- | --- | --- |
|  | **Average relative expression log_2_** | | |  | | **Percentage of positive cells** | | | | | |  | **Average relative expression log_2_** | | | | | |  | | **Percentage of positive cells** | | | | | |
| **Gene** | **A1** | **A2** | **A3** |  | | **A1** | | **A2** | | **A3** | |  | **B1** | | **B2** | | **B3** | |  | | **B1** | | **B2** | | **B3** | |
| All cells |  |  |  |  | 81 | | 75 | | 77 | |  | | |  | |  | |  | |  | | 137 | | 80 | | 75 |
| *Cspg4* | 4.45 | 0.21 | 0.06 |  | 94% | | 5% | | 1% | |  | | | 0.73 | | 2.44 | | 1.64 | |  | | 21% | | 55% | | 39% |
| *Pdgfra* | 7.55 | 0.44 | 0.32 |  | 98% | | 11% | | 6% | |  | | | 1.05 | | 4.01 | | 2.75 | |  | | 20% | | 58% | | 43% |
| *Eaat1* | 4.32 | 1.71 | 7.14 |  | 88% | | 48% | | 100% | |  | | | 4.48 | | 5.59 | | 6.04 | |  | | 80% | | 91% | | 91% |
| *Glul* | 1.91 | 3.30 | 6.50 |  | 59% | | 68% | | 96% | |  | | | 4.29 | | 5.90 | | 5.98 | |  | | 77% | | 89% | | 93% |
| *Gfap* | 0.52 | 0.36 | 0.92 |  | 11% | | 9% | | 22% | |  | | | 1.07 | | 3.97 | | 2.02 | |  | | 25% | | 60% | | 41% |
| *Gfapδ* | 0.59 | 0.61 | 0.98 |  | 23% | | 23% | | 35% | |  | | | 1.33 | | 3.53 | | 1.46 | |  | | 39% | | 64% | | 41% |
| *S100b* | 1.94 | 1.43 | 1.59 |  | 74% | | 51% | | 57% | |  | | | 1.56 | | 4.17 | | 2.37 | |  | | 63% | | 93% | | 85% |
| *Nes* | 0.24 | 0.20 | 0.13 |  | 9% | | 5% | | 4% | |  | | | 0.27 | | 1.19 | | 0.47 | |  | | 7% | | 29% | | 11% |
| *Vim* | 0.98 | 0.47 | 0.58 |  | 37% | | 13% | | 21% | |  | | | 1.66 | | 6.64 | | 1.96 | |  | | 37% | | 91% | | 57% |
| *Snap25* | 2.09 | 0.71 | 0.75 |  | 63% | | 19% | | 17% | |  | | | 0.67 | | 1.29 | | 4.18 | |  | | 21% | | 34% | | 80% |
| *Aqp1* | 0.19 | 0.72 | 0.00 |  | 9% | | 32% | | 0% | |  | | | 0.07 | | 0.08 | | 5.04 | |  | | 2% | | 3% | | 81% |
| *Aqp4* | 0.99 | 0.81 | 3.87 |  | 30% | | 28% | | 81% | |  | | | 1.97 | | 3.81 | | 2.85 | |  | | 55% | | 70% | | 60% |
| *Aqp9* | 0.49 | 0.78 | 0.05 |  | 16% | | 24% | | 3% | |  | | | 0.06 | | 0.19 | | 6.98 | |  | | 2% | | 6% | | 93% |
| *Clcn2* | 0.91 | 0.67 | 1.08 |  | 27% | | 19% | | 26% | |  | | | 0.91 | | 1.71 | | 1.35 | |  | | 26% | | 38% | | 32% |
| *Hcn1* | 1.02 | 1.29 | 0.88 |  | 25% | | 28% | | 19% | |  | | | 0.18 | | 0.87 | | 9.16 | |  | | 4% | | 20% | | 100% |
| *Hcn2* | 1.90 | 3.27 | 0.41 |  | 58% | | 65% | | 14% | |  | | | 0.86 | | 3.62 | | 9.03 | |  | | 29% | | 66% | | 97% |
| *Hcn3* | 0.93 | 1.27 | 0.68 |  | 35% | | 39% | | 22% | |  | | | 0.30 | | 1.01 | | 6.81 | |  | | 13% | | 40% | | 92% |
| *Hcn4* | 1.32 | 1.45 | 1.49 |  | 58% | | 60% | | 69% | |  | | | 0.82 | | 1.66 | | 1.93 | |  | | 40% | | 78% | | 28% |
| *Trpv4* | 0.91 | 0.53 | 1.17 |  | 30% | | 17% | | 38% | |  | | | 0.72 | | 1.71 | | 1.30 | |  | | 28% | | 45% | | 36% |
| *Grik1* | 2.31 | 0.68 | 0.88 |  | 65% | | 21% | | 23% | |  | | | 0.73 | | 2.23 | | 2.33 | |  | | 23% | | 59% | | 64% |
| *Grik2* | 2.81 | 1.17 | 0.51 |  | 84% | | 36% | | 17% | |  | | | 0.89 | | 3.16 | | 1.58 | |  | | 28% | | 74% | | 49% |
| *Grik3* | 2.91 | 0.54 | 0.65 |  | 78% | | 16% | | 14% | |  | | | 0.40 | | 2.46 | | 1.31 | |  | | 15% | | 59% | | 35% |
| *Grik4* | 1.29 | 0.25 | 0.19 |  | 47% | | 8% | | 5% | |  | | | 0.21 | | 1.31 | | 0.39 | |  | | 8% | | 38% | | 15% |
| *Grik5* | 3.57 | 1.02 | 1.91 |  | 85% | | 39% | | 56% | |  | | | 1.97 | | 4.90 | | 3.19 | |  | | 61% | | 90% | | 72% |
| *Grin3a* | 2.83 | 0.38 | 0.91 |  | 79% | | 15% | | 22% | |  | | | 0.58 | | 1.85 | | 1.61 | |  | | 21% | | 53% | | 52% |
| *Gria1* | 0.76 | 0.25 | 0.27 |  | 35% | | 11% | | 9% | |  | | | 0.16 | | 0.96 | | 0.72 | |  | | 7% | | 29% | | 28% |
| *Gria2* | 5.43 | 1.61 | 2.31 |  | 98% | | 41% | | 48% | |  | | | 2.86 | | 3.97 | | 4.18 | |  | | 66% | | 81% | | 79% |
| *Gria4* | 2.71 | 1.10 | 0.88 |  | 75% | | 40% | | 23% | |  | | | 0.86 | | 3.20 | | 1.61 | |  | | 28% | | 68% | | 44% |
| *Grin1* | 0.37 | 0.62 | 0.72 |  | 15% | | 27% | | 25% | |  | | | 0.40 | | 1.51 | | 1.32 | |  | | 18% | | 59% | | 51% |
| *Grin2a* | 2.04 | 2.92 | 2.08 |  | 53% | | 65% | | 40% | |  | | | 1.81 | | 3.40 | | 8.80 | |  | | 42% | | 61% | | 99% |
| *Grin2b* | 1.52 | 1.18 | 0.86 |  | 53% | | 35% | | 21% | |  | | | 1.13 | | 2.79 | | 2.27 | |  | | 35% | | 61% | | 61% |
| *Grin2c* | 0.41 | 0.27 | 1.09 |  | 15% | | 13% | | 34% | |  | | | 0.99 | | 1.05 | | 0.98 | |  | | 32% | | 34% | | 36% |
| *Grin2d* | 0.11 | 0.43 | 0.16 |  | 5% | | 13% | | 5% | |  | | | 0.12 | | 0.22 | | 3.17 | |  | | 5% | | 6% | | 67% |
| *Gria3* | 4.91 | 2.75 | 0.30 |  | 91% | | 56% | | 6% | |  | | | 1.02 | | 3.66 | | 9.66 | |  | | 26% | | 71% | | 100% |
| *Grm1* | 0.90 | 0.85 | 0.72 |  | 26% | | 27% | | 19% | |  | | | 0.91 | | 1.67 | | 1.40 | |  | | 28% | | 43% | | 32% |
| *Grm3* | 0.61 | 0.98 | 2.22 |  | 19% | | 28% | | 51% | |  | | | 1.69 | | 2.81 | | 1.97 | |  | | 47% | | 65% | | 43% |
| *Grm5* | 1.40 | 1.47 | 0.27 |  | 57% | | 49% | | 8% | |  | | | 0.28 | | 0.92 | | 7.46 | |  | | 13% | | 36% | | 96% |
| *Kcna3* | 0.83 | 0.90 | 0.23 |  | 41% | | 39% | | 12% | |  | | | 0.40 | | 0.83 | | 6.12 | |  | | 18% | | 34% | | 91% |
| *Kcna4* | 1.10 | 1.46 | 0.91 |  | 33% | | 40% | | 21% | |  | | | 0.68 | | 2.15 | | 1.54 | |  | | 18% | | 53% | | 39% |
| *Kcna5* | 0.92 | 0.94 | 0.90 |  | 25% | | 31% | | 26% | |  | | | 0.86 | | 2.99 | | 1.39 | |  | | 28% | | 71% | | 40% |
| *Kcnj10* | 1.82 | 1.18 | 1.33 |  | 89% | | 48% | | 62% | |  | | | 1.57 | | 3.28 | | 1.96 | |  | | 72% | | 91% | | 88% |
| *Kcnj16* | 2.58 | 0.67 | 2.13 |  | 70% | | 20% | | 49% | |  | | | 1.85 | | 3.15 | | 2.47 | |  | | 50% | | 65% | | 60% |
| *Kcnj2* | 0.47 | 0.52 | 0.44 |  | 15% | | 16% | | 12% | |  | | | 0.53 | | 2.25 | | 0.99 | |  | | 18% | | 50% | | 31% |
| *Kcnk1* | 0.74 | 0.28 | 1.00 |  | 26% | | 8% | | 30% | |  | | | 0.48 | | 0.68 | | 0.98 | |  | | 19% | | 21% | | 29% |
| *Kcnk10* | 1.60 | 0.36 | 0.38 |  | 67% | | 17% | | 13% | |  | | | 0.65 | | 1.49 | | 0.79 | |  | | 31% | | 58% | | 32% |
| *Kcnk2* | 1.31 | 0.18 | 0.25 |  | 41% | | 5% | | 8% | |  | | | 0.26 | | 1.03 | | 1.41 | |  | | 10% | | 31% | | 43% |
